# Interaction of Bile Salts With Lipid Bilayers: An Atomistic Molecular Dynamics Study

**DOI:** 10.3389/fphys.2019.00393

**Published:** 2019-04-09

**Authors:** Maria C. Neves, Hugo A. L. Filipe, Rita Leones Reis, João P. Prates Ramalho, Filipe Coreta-Gomes, Maria J. Moreno, Luis M. S. Loura

**Affiliations:** ^1^Departamento de Química, Faculdade de Ciências e Tecnologia, Universidade de Coimbra, Coimbra, Portugal; ^2^Centro de Química de Coimbra, Coimbra, Portugal; ^3^Centro de Neurociências e Biologia Celular, Universidade de Coimbra, Coimbra, Portugal; ^4^Departamento de Química, Escola de Ciências e Tecnologia, Universidade de Évora, Évora, Portugal; ^5^Centro de Química de Évora e Centro Hercules, Universidade de Évora, Évora, Portugal; ^6^QOPNA and LAQV-REQUIMTE, Departamento de Química, Universidade de Aveiro, Aveiro, Portugal; ^7^Faculdade de Farmácia, Universidade de Coimbra, Coimbra, Portugal

**Keywords:** bile salts, molecular dynamics simulations, membrane translocation, passive permeation, cholesterol absorption

## Abstract

Bile salts (BS) are biosurfactants crucial for emulsification and intestinal absorption of cholesterol and other hydrophobic compounds such as vitamins and fatty acids. Interaction of BS with lipid bilayers is important for understanding their effects on membranes properties. The latter have relevance in passive diffusion processes through intestinal epithelium such as reabsorption of BS, as well as their degree of toxicity to intestinal flora and their potential applications in drug delivery. In this work, we used molecular dynamics simulations to address at the atomic scale the interactions of cholate, deoxycholate, and chenodeoxycholate, as well as their glycine conjugates with POPC bilayers. In this set of BS, variation of three structural aspects was addressed, namely conjugation with glycine, number and position of hydroxyl substituents, and ionization state. From atomistic simulations, the location and orientation of BS inside the bilayer, and their specific interactions with water and host lipid, such as hydrogen bonding and ion-pair formation, were studied in detail. Membrane properties were also investigated to obtain information on the degree of perturbation induced by the different BS. The results are described and related to a recent experimental study (Coreta-Gomes et al., 2015). Differences in macroscopic membrane partition thermodynamics and translocation kinetics are rationalized in terms of the distinct structures and atomic-scale behavior of the bile salt species. In particular, the faster translocation of cholate is explained by its higher degree of local membrane perturbation. On the other hand, the relatively high partition of the polar glycine conjugates is related to the longer and more flexible side chain, which allows simultaneous efficient solvation of the ionized carboxylate and deep insertion of the ring system.

## Introduction

Bile salts (BS) are amphiphilic molecules synthesized in the liver and secreted into the intestinal lumen, that are involved in several biological functions such as, emulsification of hydrophobic compounds (Monte et al., 2009), signaling, metabolic and inflammatory regulation (Hylemon et al., 2009); and antibacterial activity (Urdaneta and Casadesús, 2017).

Primary BS are synthesized from cholesterol in hepatocytes, in a complex sequence of enzymatic reactions that lead to oxidation of cholesterol aliphatic side-chain to a carboxylic group and addition of hydroxyl groups to the ring system (Russell, 2003). The major primary BS in humans are chenodeoxycholate (CDCA) with 2 hydroxyl groups, and cholate (CA) with 3 hydroxyl groups. The carboxyl group of BS is usually conjugated with glycine or taurine, leading to an increase in their acidity (decrease in *pK*_a_ from about 5 to 4 and below 2, for conjugation with glycine and taurine, respectively) and therefore to an increase in the fraction of the ionized form (Carey and Small, 1972; Fini et al., 2002).

Contrary to most common surfactants that have a polar head and hydrophobic tail, BS have two surfaces, one hydrophilic and the other hydrophobic. This property leads to the formation of very small disc shaped micelles, typically 4 to 6 BS molecules *per* micelle for the trihydroxy BS and 10 to 20 for the dihydroxy BS (Hofmann and Small, 1967). Hydrophobic solutes interact efficiently with the non-polar core of the micelles, and this step is essential for their emulsification and absorption at the intestine (Woollett et al., 2006; Coreta-Gomes et al., 2012, 2016).

When in the presence of intestinal microbiota, BS are deconjugated and/or suffer biotransformations originating secondary BS such as deoxycholate (DCA), among others (Floch, 2002). About 90% of the BS secreted into the intestine are reabsorbed. This occurs actively through the action of BS transporters (Alrefai and Gill, 2007), and through passive permeation of the unconjugated form of the BS down the concentration gradient (Donovan and Jackson, 1997; Floch, 2002; Coreta-Gomes et al., 2015).

Detailed knowledge of the interaction of BS with dietary lipids and lipid membranes at molecular level is crucial to understand their transport from the liver and storage at the gallbladder, as well as the discharge and reabsorption in the intestine, shedding light on their role in health and disease states. Moreover, due to the crucial action on the absorption of hydrophobic nutrients and drugs, it is important to characterize their partition and effects on the barrier properties of biological membranes. In particular, the protective effect of some trihydroxy BS, compared to the toxicity of less polar BS, may be related with their affinity for the lipid bilayer, and perturbation of membrane barrier properties (Heuman et al., 1991; Mello-Vieira et al., 2013; Coreta-Gomes et al., 2015).

Computational techniques have become valuable tools to study biomolecular assemblies, and indeed several simulation studies of BS both dispersed in aqueous media and in the presence of phospholipids have been reported, using atomistic molecular dynamics (Marrink and Mark, 2002; Pártay et al., 2007; Mustan et al., 2015). This type of simulations can provide considerable detail regarding location, orientation, and characterization of preferred interaction of BS molecules with lipids, as well as local perturbations induced by the former on bilayer properties, which in turn may relate to differences in the behavior of the distinct BS when interacting with membranes. Previous studies were focused on the properties of pure BS micelles and mixed micelles formed at high BS/lipid ratios, and only the ionized forms of the BS were considered. They have shown that at low BS concentrations primary micelles are kept together by hydrophobic interactions, while at high concentrations the formation of larger, secondary micelles is promoted via hydrogen-bonding interactions. Experimental results at low BS/lipid ratios have shown that at low local concentrations in the lipid membrane, the neutral form of the BS is stabilized and may become the most abundant at physiological conditions for unconjugated BS (Coreta-Gomes et al., 2015). Also, significant differences were encountered for the rate of permeation of di- and trihydroxy BS through POPC bilayers in those low, non-lytic BS/lipid ratios. The understanding of the molecular interactions established by the distinct BS with the lipid bilayer at non-lytic conditions is of high relevance to rationalize and predict the effect of BS on the barrier properties of the intestinal membrane at physiological conditions. It may also allow understanding the molecular properties of the BS that lead to toxic and protective effects, with impact on the design of new non-toxic agents to enhance the bioavailability of pharmacological agents.

**FIGURE 1:**
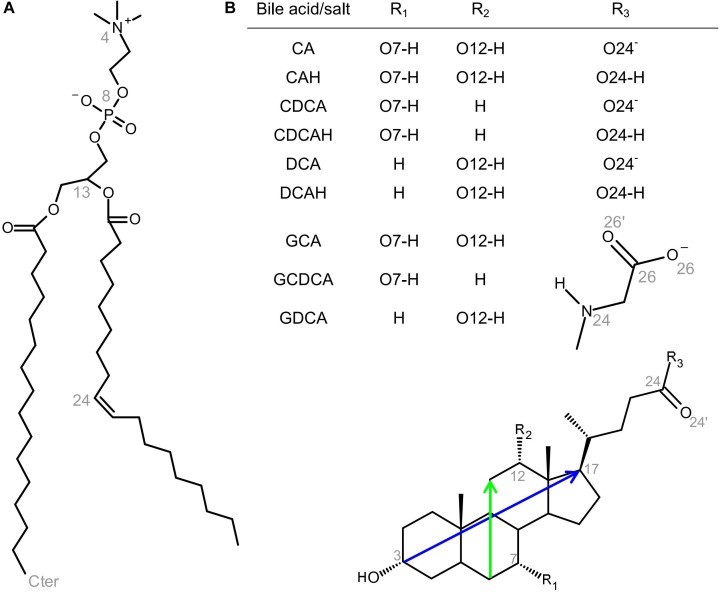
Structures of POPC **(A)** and BS addressed in this study (**B**; see also inset table for definition of R_1_, R_2,_ and R_3_ for the different BS species), showing the numbering of selected atoms pertinent to the simulations, as well as the definition of the long and short molecular axes of BS (blue and green, respectively).

In this work, we report atomistic MD simulations of CA, CDCA, and DCA BS molecules, both in their ionized (basic) and non-dissociated (acid, here designated as CAH, CDCAH, and DCAH, respectively) species, as well as the ionized form of their glycine conjugates (GCA, GCDCA, and GDCA), interacting with a 1-palmitoyl-2-oleoyl-*sn*-glycero-3-phosphocholine (POPC) bilayer (see Figure 1 for structures and numbering of relevant atoms). Both ionization states were considered for the unconjugated BS, because even though the basic species are expected to be dominant in aqueous media [*pK*_a_ between 4.5 and 5.1 at 25°C (Moroi et al., 1992)], the substantially more lipophilic protonated forms interacts preferably with lipid bilayers leading to an increase in the apparent *pK*_a_ with the protonated form being significant even at neutral pH (Coreta-Gomes et al., 2015). Simulations were done at a relatively low BS/POPC molecular ratio (2:128), allowing to characterize and distinguish the interaction between the BS and the membrane based on their different molecular structures.

## Materials and Methods

All simulations were carried out with Gromacs versions 4 (Pronk et al., 2013) and 5 (Abraham et al., 2015). The topology of the POPC molecule (Figure 1A) consisted of a united-atom description for CH, CH_2_, and CH_3_ groups, based on the parameters presented by Berger et al. (1997) for 1,2-dipalmitoyl-*sn*-glycero-3-phosphatidylcholine (DPPC), and by (Bachar et al., 2004) for the acyl chain *cis*-double bonds. While this force field has been put into question, mostly in regarding to its overcondensation of bilayers with high cholesterol content, it predicts accurate values of average area/lipid and acyl chain order parameter profiles in pure POPC (Ferreira et al., 2013; Botan et al., 2015). The structures and topologies of unconjugated BS in both protonated and ionized forms, as well as the ionized glycine-conjugated derivative (Figure 1B), were adapted from that of cholesterol as used by Höltje et al. (2001) (available for download at the GROMACS webpage^1^), by adding the missing hydroxyl, amide and carboxylic groups to the tetracyclic ring system and side-chain, using standard force field parameters (Jorgensen et al., 1983; Van Gunsteren and Berendsen, 1987; Van Buuren and Berendsen, 1993; Van Buuren et al., 1993; Mark et al., 1994; Liu et al., 1995). Partial atomic charges of BS were calculated from the optimized geometries obtained from quantum chemical calculations performed at the Hartree–Fock level and the 6–31+G(d) basis set, followed by a least-squares fit to the electrostatic potential obtained at the same theory level, according to the Kollman and Singh scheme (Singh and Kollman, 1984; Besler et al., 1990). All quantum chemical calculations were performed using the GAMESS-US package (Schmidt et al., 1993; Gordon and Schmidt, 2005). The simple point charge water model was used (Berendsen et al., 1981).

Using standard GROMACS tools, a fully hydrated POPC bilayer (128 POPC: 5412 H_2_O) was built. Subsequently, two BS molecules were added. For each composition, two simulations were carried out, with initial BS location either in the aqueous medium or in the bilayer center with the ring system parallel to the bilayer plane. For the simulations with charged BS species, two sodium ions were randomly added to the aqueous medium to ensure electroneutrality.

All systems were simulated under *NpT* conditions at 298.15 K and 1 bar. Equilibration/ production run protocols and other simulation options were as described elsewhere (Filipe et al., 2015). The pure POPC system was simulated for 100 ns, of which the last 50 ns were used for calculation of reference area/lipid, atomic positions and deuterium order parameter profiles. For the simulations in which the solutes were placed in the bilayer center at the start, they moved to their equilibrium transverse location within the first 50 ns of the 200 ns production run, and the last 150 ns were taken for analysis. However, in some of the systems where BS molecules were initially put in the aqueous medium/membrane interface, very slow convergence to the equilibrium position was observed, occurring in a time-scale of 500–1000 ns. For the unconjugated BS species, we extended the original 200 ns simulations until the solute molecules reached equilibrium position and orientation identical to those observed in the corresponding simulation with the BS molecules initially in the center of the bilayer, and maintained such stable transverse location for >200 ns. The final 150 ns of these simulations were taken into account for analysis, and all parameters shown were calculated averaging over the two simulations. For the conjugated BS, the least hydrophobic species, convergence of simulations where the molecules started in the aqueous medium/membrane interface may require prohibitively long simulations in some cases. For those systems, only the simulations where the BS started from center of the bilayer were used for analysis. In any case, as shown in Supplementary Figures S1, S2, in the simulations where the BS molecules were initially placed in the water medium, most of them were able to insert in the equilibrium position in the membrane interface by the end of the simulation (see Supplementary Figure S2). For visualization of structures and trajectories, VMD software was used (Humphrey et al., 1996). Standard errors of the average values were calculated using the block method of Flyvbjerg and Petersen (Flyvbjerg and Petersen, 1989).

Calculated logarithms of octanol/water partition coefficients (CLogP) were obtained using Marvinsketch 15.10.26, 2015, ChemAxon^2^.

## Results and Discussion

### Localization and Orientation From Atomistic MD

**FIGURE 2:**
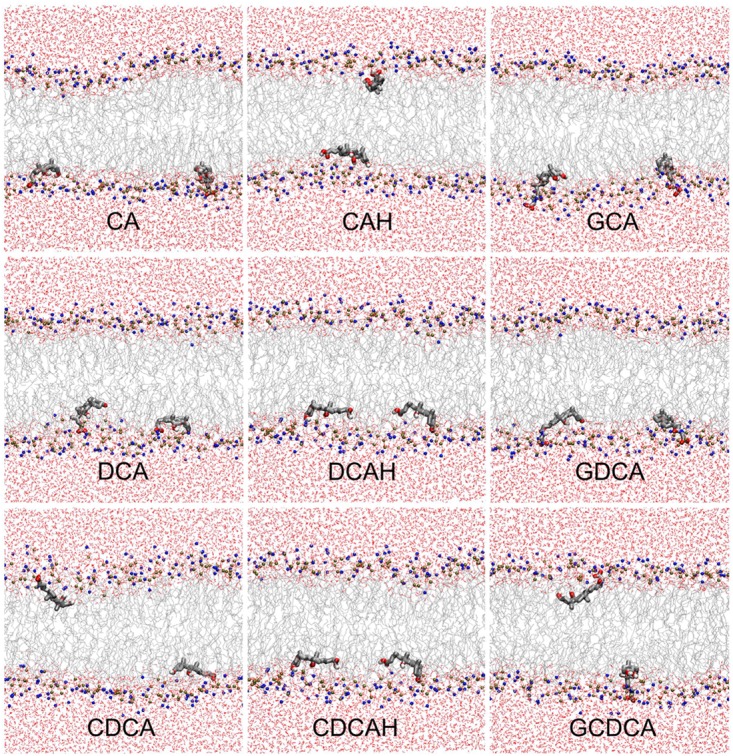
Final snapshots of the simulations with initial BS location in the center of the bilayer. BS molecules are depicted in licorice, while POPC and water molecules are depicted in line style. Atom colors are gray, red, brown, blue, and white, for C, O, P, N, and H, respectively.

As described in the methods section, two sets of simulations were performed; one where the BS molecules were initially placed in the aqueous media (*z*∼2–2.5 nm) and another with the BS molecules initially placed in the center of the bilayer (*z* = 0 nm). Equivalent localizations were obtained from both sets at the end of the simulations, indicating that the positions obtained correspond to the equilibrium position. Figure 2 shows the final configurations obtained in the simulations containing two BS molecules initially located in the center of the POPC membrane. These snapshots illustrate that all studied BS species are typically located near the carbonyl/glycerol region of the bilayer, corresponding to region 2 of the Four-Region bilayer model, where water density drops to <1% (Marrink and Berendsen, 1994; Disalvo et al., 2014). The BS molecules mostly orient parallel to the membrane plane, with the hydrophobic face oriented to the center of the membrane. The final snapshots for all simulations, as well as the time evolution in the position of BS center of mass (COM), are shown in the Supplementary Figures S1, S2.

**FIGURE 3:**
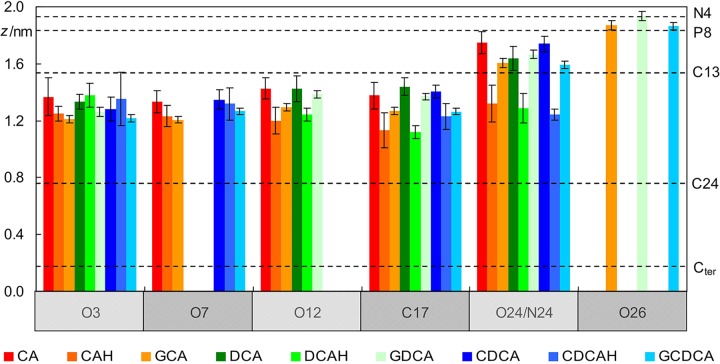
Average location of selected POPC (dashed horizontal lines; please refer to Figure 1 for numbering) and BS atoms (vertical bars) during the simulations (see Figure 1 for atom numbering).

A more detailed description of the behavior of the BS molecules is revealed by inspecting the locations of individual BS atoms, in comparison with those of POPC (Figure 3). It is apparent that the hydroxyl O atoms of all BS species lie at a similar depth in the bilayer, at ∼1.3 ± 0.2 nm, just below the POPC C13 atom (and at a similar location to that of the POPC ester O atoms). This is valid for protonated, ionized and glycine-conjugated BS. From a deeper inspection of Figure 3 for the case of unconjugated ionized BS, it is observed that the hydroxyl groups nearer to the polar carboxylic group (O12) are located closer to the water medium for CA and DCA. This is a consequence of the charged carboxylate group having an external position just below the POPC headgroup atoms (see O24 in Figure 3). This also affects the positions of the ring system atoms where the side chain is attached, as visible in the positions of the C17 atoms. At variance, the neutral carboxylic groups have average locations very similar to those of the hydroxyl atoms, and for the uncharged BS species, C17 has a slightly deeper location than the other investigated atoms.

Regarding the glycine conjugates, the hydroxyl groups have similar locations to those of the unconjugated species. The locations of O3 and O7 are slightly deeper, whereas those of O12 (as well as C17) are intermediate between the ionized and non-ionized forms of the unconjugated forms. However, these differences fall within the estimated uncertainty. On the side chain, the amide N24 atoms of the conjugated species have average transverse locations comparable to those of the O24 charged carboxylate atom of the ionized unconjugated BS, and therefore significantly shallower than the neutral carboxylic group of the unionized unconjugated species. However, the most external atoms of the glycine conjugates are those of the charged carboxylate (O26 in Figure 3), which on average are located in the headgroup region of the bilayer, clearly outside the carboxylate O24 atoms of GCA, DCA, and CDCA. This more external location of the charged end of the side chain of glycine conjugates, compared to the unconjugated BS species, is probably the result of two effects: (i) the higher conformational flexibility of the longer side-chain; and (ii) the existence of another polar moiety (the amide group), to provide additional anchoring to the lipid/water interface.

**FIGURE 4:**
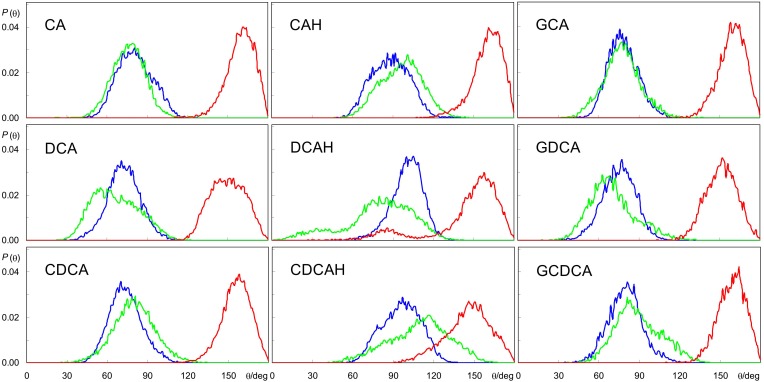
Angular distributions for the tilt of BS long and short axes (blue and green, respectively), and normal to the plane defined by these axes, relative to the bilayer normal (red) during the simulations (see Figure 1 for axis definition).

The similar hydroxyl locations of all species is the result of the BS ring system preferentially aligning roughly parallel to the bilayer plane, as shown in Figure 2, and as illustrated in the angular distributions of the long and short axes tilts, relative to the bilayer plane normal, Figure 4 (for definition of axes, see Figure 1). The distributions of both axes are wide and sometimes affected by the statistical limitations arising from the small number of molecules (e.g., the tail observed for the orientation of DCAH short axes in the small angle region). In any case, the angle distributions are mostly centered near ∼90°, corresponding to a preferential orientation perpendicular to the bilayer normal. Correspondingly, the normal to the approximate ring system plane (note that the ring system is not strictly planar) is almost parallel to the bilayer normal (tilt values close to 180°). Despite the rather similar orientation pattern for all studied species, there are identifiable subtle dissimilarities, related to the differences in their molecular structure. For example, the displacement of the short axis of the distributions to smaller angles in the different forms of deoxycholate (DCA, DCAH, and GDCA) is a result of the absence of a hydroxyl group attached to C7. On the other hand, it is apparent that for the protonated species the tilt distributions of both axes are slightly displaced to higher values (mostly θ > 90°) compared to the ionized species (mostly θ < 90°). These minor differences in orientation stem from the more external location of the carboxylate group in the ionized species as commented above. Finally, the tilt distributions of the normal to the plane defined by the long and short axes is generally slightly closer to 180° for the trihydroxy BS compared to the dihydroxy derivatives, probably because the additional OH group helps to keep the ring system more firmly parallel to the bilayer plane.

The localization of the BS in the lipid bilayer can be used to rationalize the relative stability of the neutral (protonated) and ionized (deprotonated) forms when interacting with POPC bilayers. The neutral form of unconjugated BS is stabilized by 14 kJ/mol (Δ*pK_a_* = 2.5) (Coreta-Gomes et al., 2015). This correlates well with the ∼4 Å deeper localization of the carboxylic group when compared with that of the carboxylate (Figure 3), which permits a deeper localization of the non-polar face of the BS (C17 located at 1.1–1.2 and 1.4 Å for the protonated and deprotonated species, respectively). In addition, the water density decreases very significantly in this region of the membrane (Filipe et al., 2015) leading to stabilization of the system due to a decrease in the contribution of the hydrophobic effect. For glycine conjugates the ionized species are always dominant, the neutral species being negligible. Glycine conjugation leads to a larger distance between the non-polar surface of the BS and the carboxylate group (Figure 1), allowing the localization of the charged group at a more external position (Figure 3), while maintaining the non-polar groups in a bilayer region with a relatively small water density.

The localization and orientation of the BS in the bilayer influences the relative stability of the molecules and therefore their affinity for the bilayer. The discussion regarding the relative partition coefficients will be addressed in the following section, because they are also influenced by the interactions established between the BS and the lipids.

### Interactions Between BS and Lipid or Water

Bile salts location and orientation in the bilayer reflect their ability to establish strong interactions with both lipid and water molecules, particularly those driven by polarity and hydrogen bonding capacity.

**FIGURE 5:**
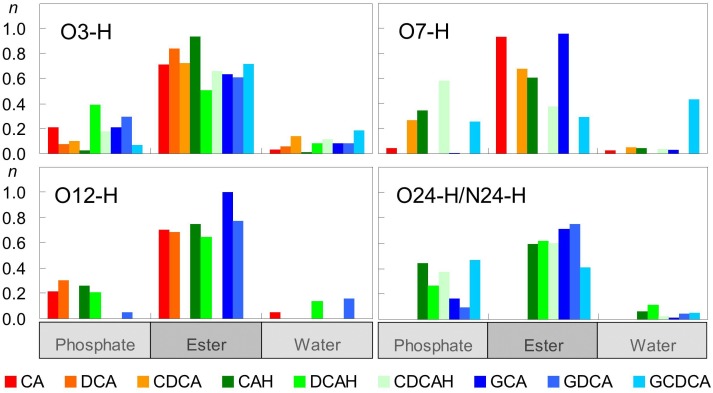
Average number of hydrogen bonds per BS molecule between BS hydroxyl/carboxyl/amide donor groups and different water and lipid acceptor atoms.

Figure 5 shows computed average numbers of H-bonds between BS OH groups and POPC O atoms. The first observation is that, with the sole exception of O7-H group of GCDCA, OH groups from the BS interact much more frequently with O acceptor groups from POPC than with water. Among the interactions established with the lipid, H-bonds to carbonyl/ester atoms are generally more probable than to phosphate oxygens, reflecting the preferential location of the hydroxyl donor groups in the glycerol region rather than near the lipid headgroups. Overall, all hydroxyl groups are continuously involved in H-bonding.

**FIGURE 6:**
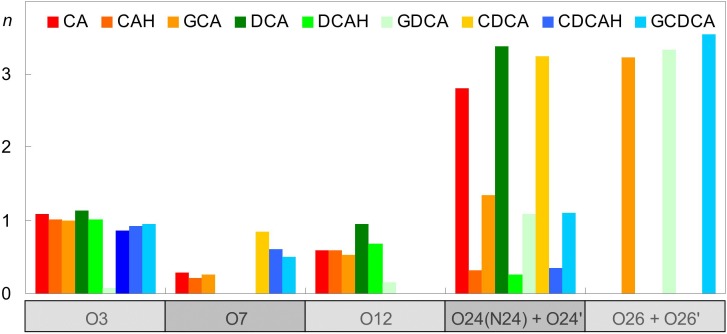
Average number of hydrogen bonds per BS between water donors and BS acceptor atoms.

Besides serving as donors to water or lipid atoms, oxygen atoms of BS (both hydroxyl and carboxyl/carboxylate) can also act as H-bonding acceptors. Given the absence of H-bonding donor groups in the POPC molecule, those interactions can only be established with water. This type of bonds is especially probable for the less sterically hindered O3 atom in all BS species, with the exception of GDCA, and the carboxylate oxygens in the ionized forms (Figure 6). The latter are particularly probable, as expected from the highly electronegative character of carboxylate O atoms and their external locations. Amide atoms of the glycine conjugates form H-bonds with water less frequently than those of carboxylate groups, because of their slightly more internal position, the lower electronegative character of the N atom, and its decreased steric accessibility to water.

**FIGURE 7:**
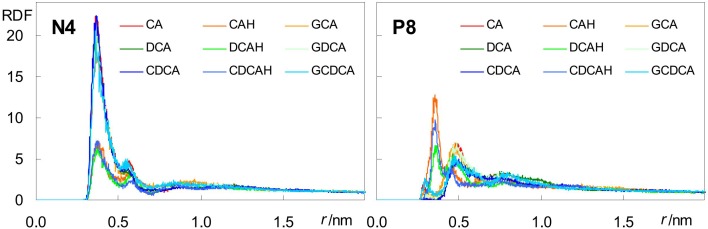
Radial distribution functions (RDFs) of POPC N4 (left) and P8 (right) atoms around BS carboxylate O atoms.

Besides H-bonding from water, and consistent with their external location, negatively charged carboxylate groups can also form close contacts with cationic POPC choline groups. This can be verified in the radial distribution functions (RDFs) of both POPC N4 and P8 atoms around carboxylic/carboxylate O atoms (Figure 7).

For the anionic species CA, DCA, and CDCA (as well as their glycine conjugates), RDFs of POPC N4 (Figure 7, left) show a well defined peak at *r* ∼0.35–0.40 nm, reflecting formation of an ion pair between the anionic carboxylate and the cationic -N(CH_3_)_3_ choline groups. Crucially, this peak is significantly reduced in the unconjugated neutral species, confirming the electrostatic nature of this interaction. On the other hand, RDFs of P8 (Figure 7, right) around the carboxylate are poorly structured and typically displaced to larger *r*-values, reflecting absence of specific favorable interaction for all ionized BS species. Conversely, the corresponding peaks are clearly visible in the RDFs of P8 around the neutral carboxylic groups, reflecting the increased frequency of H bonding from BS carboxylic group, which is absent in the ionized species, to lipid phosphate O atoms.

Altogether, these results help to explain both the similar positions of the hydroxilic OH groups (which predominantly form H-bonds with lipid ester in all cases) and the differences in the positions of the carboxylic/carboxylate groups (which form H-bonds with lipid oxygen atoms, or establish electrostatic pair interactions with lipid choline groups, respectively) between the protonated and ionized forms. The possibility to establish these favorable interactions determines the transverse positions of BS polar groups, and in turn affects the location and orientation of the molecule as a whole.

The extensive electrostatic and hydrogen bonding interactions established between the BS and the POPC bilayer while maintaining the non-polar surface protected from the aqueous phase explains the relatively high partition coefficients obtained experimentally for these molecules (3 × 10^2^ to 3 × 10^3^, for tri- and dihydroxy BS, respectively) (Coreta-Gomes et al., 2015; Yang et al., 2015). The smaller affinity of the trihydroxy BS for the POPC bilayer is due to their higher polarity, which leads to stronger interactions with the aqueous phase (CLogP = 2.5 and CMC = 16 mM for CAH and CLogP = 3.8 and CMC = 8 mM for DCA) (Roda et al., 1983). It is observed experimentally that conjugation with glycine does not lead to a decrease in the affinity for lipid bilayers nor in their CMC despite the increased general polarity (CLogP = 2.7 for GDCA). As shown in Figure 3, the conjugation with glycine leads to a deep localization of the ring system hydroxyl groups, and a shallower localization of the charged carboxylate. Thus, the increased spacing distance between the non-polar surface of the BS and the charged carboxylate allows fulfilling of BS favorable interactions with both the membrane and water.

The extensive network of hydrogen bonds and electrostatic interactions between the BS and the membrane is also reflected in the thermodynamic parameters for partition between the aqueous phase and the lipid bilayers. Indeed, a large and negative value is obtained for the enthalpy contribution $\left(\Delta H_{p}^{o}\right)$ associated with partition of the BS to a POPC bilayer (Coreta-Gomes et al., 2015). In addition, a larger enthalpy contribution is observed for the unconjugated when compared with glycine-conjugated BS (-25 kJ/mol for DCA, compared to -15 kJ/mol for GDCA, and -22 kJ/mol for CDCA, compared to -16 kJ/mol for GCDCA). The amide group present in the glycine conjugated BS is able to establish one additional H-bond as donor to the ester/phosphate of POPC and an additional H-bond as acceptor to water, when compared with the corresponding unconjugated BS. However, the enthalpy variation upon association with the membrane reflects the balance between the formed and broken interactions. The smaller enthalpy stabilization observed for the glycine conjugates should reflect a relative decrease in the total number of hydrogen bonds, when going from the aqueous phase into the membrane, caused by the placement of the amide group in a membrane location with a relatively small water density.

### Effect of BS on Lipid Properties

**FIGURE 8:**
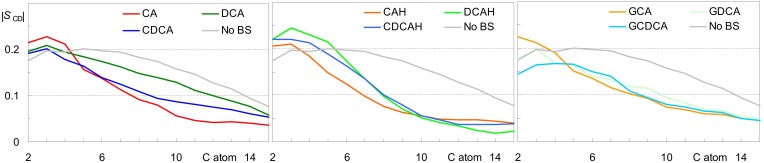
Deuterium order parameter profiles for the palmitoyl chain of POPC for all lipids in the absence of BS (black) and for lipid chains located laterally within 0.6 nm of the COM of a BS molecule. Left, middle and right panels refer to systems with inserted anionic unconjugated, neutral unconjugated and conjugated BS, respectively.

Average area/lipid and bilayer thickness values close to those obtained for pure POPC (0.66 ± 0.02) nm^2^ and (3.66 ± 0.10) nm, respectively (Filipe et al., 2015) were obtained for all studied systems, with differences falling within the estimated uncertainty. Similarly, only very slight variations were observed in calculated deuterium order parameter profiles compared to those pure POPC bilayers (not shown). The slight extents of these effects were expected, because of the low BS concentration in the studied systems. However, they do not exclude the possibility of significant local BS-induced perturbation. For this reason, we calculated the lipid chain order parameters averaging over the POPC acyl chains closest (<0.6 nm) to each of the BS molecules. As shown in Figure 8, some ordering is generally apparent in the upper segments of the POPC acyl chains, corresponding to the region of the bilayer where the BS molecules are located. However, from the 5th–6th carbon onward, local order parameter values are lower than those calculated in the absence of BS. One possible reason for this reduction in the order of the innermost lipid acyl chain segments probably is the superficial location of BS molecules in the bilayer, leaving free space underneath them, which is filled by disordered lipid chains. The effect is more significant for cholic acid for both ionization states. It is noteworthy that for the dihydroxy BS, the protonated form (Figure 8, middle) induces a more significant decrease in the lipid local order parameter than the corresponding ionized form (Figure 8, left and middle). This is due to the location and orientation of the BS, being the ionized BS more aligned with the lipids (and hence less perturbing) due to the more external location of the charged carboxylate. Regarding the glycine conjugates, the most significant observation is the similar effect of di- and trihydroxyl BS on the POPC order parameter from the 5th carbon onward (Figure 8, right).

**FIGURE 9:**
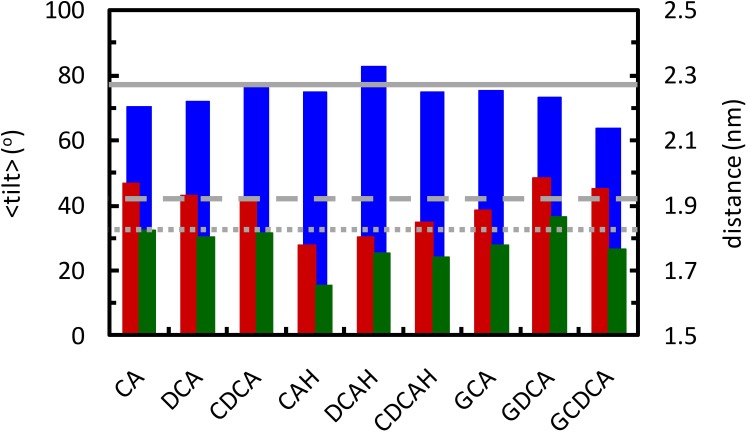
Average tilt angle of the lipid P-N vector (blue, left ordinate axis), and average position of N and P atoms (red and green, respectively; right ordinate axis), for the lipids that are within 0.6 nm from the BS molecules. Solid, dashed and dotted gray lines show the average tilt angle and the position of N and P atoms in the pure POPC bilayer, respectively.

Possible perturbation of the bilayer due to the presence of the BS may also be evaluated from the position of the phosphate (P) and choline (N) groups, as well as from the angle between the P-N vector and the bilayer normal (P-N tilt). In fact, changes in the P-N tilt may be originated from alterations in the relative position of the P and/or N groups. The distributions of these parameters are shown in Supplementary Figures S3, S4 for POPC molecules with a BS solute at close (*R* < 0.6 nm) or intermediate (0.6 nm < *R* < 1.2 nm) distance *R*, respectively, and compared to those obtained for pure phospholipid bilayers. For *R* < 0.6 nm, average values for these parameters are shown in Supplementary Table S1 and displayed in Figure 9.

Inspection of Figure 9 reveals that POPC molecules in the vicinity of ionized BS species, either conjugated or non-conjugated, have invariant (CDCA, GCA) or reduced (CA, DCA, GDCA, and GCDCA) average P-N tilt angles. This is typically achieved by a modest lowering of the lipid phosphate group, concomitant with a slightly higher position of the choline. The latter may be understood in terms of the external location of the carboxylate group and the ∼0.4 nm distance required for the choline-carboxylate ion pair (Figure 7, left), which is particularly evident for the glycine conjugates. This effect is absent in the protonated unconjugated series (CAH, DCAH, and CDCAH). For these molecules, the P-N tilt angle is essentially unaltered (or slightly increased for DCAH), which is the result of a considerably deeper location of both POPC choline and phosphate groups, probably due to hydrogen bonding between the carboxylic group and phosphate O atoms (Figure 5, bottom right panel). A more detailed discussion of these variations is provided in the Supplementary Material.

From the overall analysis of the effects of the distinct BS on the properties of both the POPC headgroup and acyl chains, some global observations may be made: (i) unconjugated BS with 3 hydroxyl groups lead to a much stronger local perturbation of the POPC bilayer than do unconjugated BS with 2 hydroxyl groups; (ii) the establishment of an hydrogen bond between the carboxylic group of unconjugated BS and the POPC phosphate group leads to a deeper localization of both the phosphate and the choline, thus leading to a significant decrease in the local thickness of the bilayer; and (iii) the presence of an OH group near the polar side chain, in both conjugated and unconjugated deoxycholate, helps to orient the fused rings parallel to the lipid chains, reducing membrane perturbation.

The local perturbation in the lipid bilayer induced by the BS molecules is well correlated with the observed rate of translocation for unconjugated BS. It was experimentally observed that CA translocates relatively fast through a POPC bilayer, followed by CDCA and DCA (Coreta-Gomes et al., 2015). This behavior goes in parallel with the much stronger local perturbation of CA (Figure 8). In addition, the protonated form of the unconjugated BS establishes an hydrogen bond with the phosphate group, stabilizing this BS species and leading to the increase of 2.5 units in the *pK*_a_ for the ionization of this group. To establish this interaction, the phosphate group moves into a deeper position in the bilayer, leading to a significant decrease in the local membrane thickness which further facilitates the translocation of the BS and that of other molecules in its near proximity.

The glycine-conjugated BS lead to a smaller local perturbation of the bilayer, and the discrimination between the effects of the distinct BS is less pronounced. Also, stabilization of the charged carboxylate leads to a higher energy barrier at the transition state for translocation (polar groups in the center of the bilayer). Both effects result in a slower rate of translocation.

## Conclusion

In this work, we used molecular dynamics simulations to characterize at atomic detail the interaction of the BS cholate, deoxycholate and chenodeoxycholate, as well as their ionized glycine conjugates, with POPC bilayers. The simulation results agree with previously reported experimental data (Coreta-Gomes et al., 2015), and describe with high resolution the behavior of BS molecules interacting with lipid membranes at low (non-lytic) local concentrations. The similar location of the ring system in the bilayer, allowing the water exposure of the charged group of the conjugated BS molecules while inserted in the membrane, is consistent with the similar membrane partition coefficient for conjugated and unconjugated BS. Also, the interfacial location of the molecules in the membrane where the water accessibility is reduced agrees with the similar Δ*pK*_a_ change that results in an increased fraction of all unconjugated BS species in the protonated form. On the other hand, the charged group of the glycine conjugates is more externally located in the membrane, being able to remain in a fully ionized state. The detailed characterization of the interactions between BS and lipid molecules also agrees with the intrinsic thermodynamic parameters for the partition to the membranes. The favorable partition enthalpies obtained experimentally can be explained by the formation of hydrogen bonds and electrostatic pair interactions between the BS and the lipids. The ability to increase the *pK*_a_ value of the ionizable group, conjugated with the higher local perturbation on the lipid structure allows the trihydroxy unconjugated BS to translocate faster in the lipid bilayer. Finally, neutral non-conjugated BS induce larger perturbation of nearby lipids, reducing the local bilayer thickness. This may result in a reduced membrane barrier effectiveness, which can be useful for drug-delivery applications and relevant to the physiological role of BS.

## Author Contributions

HF, MM, and LL designed the research. JPR, HF, and LL set up the simulation models. MN, HF, RR, and LL carried out the simulations and analyses. HF, FC-G, MM, and LL interpreted the results and wrote the manuscript. All authors read and approved the final manuscript.

## Conflict of Interest Statement

The authors declare that the research was conducted in the absence of any commercial or financial relationships that could be construed as a potential conflict of interest.

## Footnotes

1 http://www.gromacs.org/@api/deki/files/29/=cholesterol.tgz

2 http://www.chemaxon.com

## References

[B1] Abraham M. J. Murtola T. Schulz R. Páll S. Smith J. C. Hess B. (2015). GROMACS: high performance molecular simulations through multi-level parallelism from laptops to supercomputers. *SoftwareX* 1–2, 19–25. 10.1016/j.softx.2015.06.001

[B2] Alrefai W. A. Gill R. K. (2007). Bile acid transporters: structure, function, regulation and pathophysiological implications. *Pharm. Res.* 24 1803–1823. 10.1007/s11095-007-9289-1 17404808

[B3] Bachar M. Brunelle P. Tieleman D. P. Rauk A. (2004). Molecular dynamics simulation of a polyunsaturated lipid bilayer susceptible to lipid peroxidation. *J. Phys. Chem. B* 108 7170–7179. 10.1021/jp036981u

[B4] Berendsen H. J. C. Postma J. P. M. Gunsteren W. F. Hermans J. (1981). “Interaction Models for Water in Relation to Protein Hydration,” in *Intermolecular Forces*, ed. Pullman B. (Amsterdam: Springer), 331–342. 10.1007/978-94-015-7658-1_21

[B5] Berger O. Edholm O. Jahnig F. (1997). Molecular dynamics simulations of a fluid bilayer of dipalmitoylphosphatidylcholine at full hydration, constant pressure, and constant temperature. *Biophys. J.* 72 2002–2013. 10.1016/S0006-3495(97)78845-3 9129804 PMC1184396

[B6] Besler B. H. Merz K. M. Kollman P. A. (1990). Atomic charges derived from semiempirical methods. *J. Comput. Chem.* 11 431–439. 10.1002/jcc.540110404

[B7] Botan A. Favela-Rosales F. Fuchs P. F. J. Javanainen M. Kanduè M. Kulig W. (2015). Toward atomistic resolution structure of phosphatidylcholine headgroup and glycerol backbone at different ambient conditions. *J. Phys. Chem. B* 119 15075–15088. 10.1021/acs.jpcb.5b04878 26509669 PMC4677354

[B8] Carey M. C. Small D. M. (1972). Micelle formation by bile salts: physical-chemical and thermodynamic considerations. *Arch. Intern. Med.* 130 506–527. 10.1001/archinte.1972.03650040040005 4562149

[B9] Coreta-Gomes F. M. Martins P. A. T. Velazquez-Campoy A. Vaz W. L. C. Geraldes C. F. G. Moreno M. J. (2015). Interaction of bile salts with model membranes mimicking the gastrointestinal epithelium: a study by isothermal titration calorimetry. *Langmuir* 31 9097–9104. 10.1021/acs.langmuir.5b01810 26241730

[B10] Coreta-Gomes F. M. Vaz W. L. C. Wasielewski E. Geraldes C. F. G. Moreno M. J. (2016). Quantification of cholesterol solubilized in dietary micelles: dependence on human bile salt variability and the presence of dietary food ingredients. *Langmuir* 32 4564–4574. 10.1021/acs.langmuir.6b00723 27079626

[B11] Coreta-Gomes F. M. Vaz W. L. C. Wasielewski E. Geraldes C. F. G. C. Moreno M. J. (2012). Quantification of cholesterol solubilized in bile salt micellar aqueous solutions using 13C nuclear magnetic resonance. *Anal. Biochem.* 427 41–48. 10.1016/j.ab.2012.04.028 22569559

[B12] Disalvo E. A. Martini M. F. Bouchet A. M. Hollmann A. Frías M. A. (2014). Structural and thermodynamic properties of water–membrane interphases: significance for peptide/membrane interactions. *Adv. Coll. Interface Sci.* 211 17–33. 10.1016/j.cis.2014.05.002 25085854

[B13] Donovan J. M. Jackson A. A. (1997). Transbilayer movement of fully ionized taurine-conjugated bile salts depends upon bile salt concentration, hydrophobicity, and membrane cholesterol content. *Biochemistry* 36 11444–11451. 10.1021/bi9705927 9298964

[B14] Ferreira T. M. Coreta-Gomes F. Ollila O. H. S. Moreno M. J. Vaz W. L. C. Topgaard D. (2013). Cholesterol and POPC segmental order parameters in lipid membranes: solid state 1H-13C NMR and MD simulation studies. *Phys. Chem. Chem. Phys.* 15 1976–1989. 10.1039/c2cp42738a 23258433

[B15] Filipe H. A. L. Santos L. S. Prates Ramalho J. P. Moreno M. J. Loura L. M. S. (2015). Behaviour of NBD-head group labelled phosphatidylethanolamines in POPC bilayers: a molecular dynamics study. *Phys. Chem. Chem. Phys.* 17 20066–20079. 10.1039/c5cp01596k 26063509

[B16] Fini A. Feroci G. Roda A. (2002). Acidity in bile acid systems. *Polyhedron* 21 1421–1427. 10.1016/S0277-5387(02)00968-3

[B17] Floch M. H. (2002). Bile salts, intestinal microflora and enterohepatic circulation. *Dig. Liver Dis.* 34 S54–S57. 10.1016/S1590-8658(02)80165-7 12408441

[B18] Flyvbjerg H. Petersen H. G. (1989). Error estimates on averages of correlated data. *J. Chem. Phys.* 91 461–466. 10.1063/1.457480

[B19] Gordon M. S. Schmidt M. W. (2005). “Chapter 41 - Advances in electronic structure theory: GAMESS a decade later,” in *Theory and Applications of Computational Chemistry*, eds Dykstra C. E. Frenking G. Kim K. S. Scuseria G. E. (Amsterdam: Elsevier), 1167–1189.

[B20] Heuman D. M. Pandak W. M. Hylemon P. B. Vlahcevic Z. R. (1991). Conjugates of ursodeoxycholate protect against cytotoxicity of more hydrophobic bile salts: in vitro studies in rat hepatocytes and human erythrocytes. *Hepatology* 14 920–926. 10.1002/hep.1840140527 1937396

[B21] Hofmann A. F. Small D. M. (1967). Detergent properties of bile salts: correlation with physiological function. *Ann. Rev. Med.* 18 333–376. 10.1146/annurev.me.18.020167.002001 5337530

[B22] Höltje M. Förster T. Brandt B. Engels T. Von Rybinski W. Höltje H.-D. (2001). Molecular dynamics simulations of stratum corneum lipid models: fatty acids and cholesterol. *Biochim. Biophys. Acta* 1511 156–167. 10.1016/S0005-2736(01)00270-X 11248214

[B23] Humphrey W. Dalke A. Schulten K. (1996). VMD: visual molecular dynamics. *J. Mol. Graph.* 14 33–38. 10.1016/0263-7855(96)00018-5 8744570

[B24] Hylemon P. B. Zhou H. Pandak W. M. Ren S. Gil G. Dent P. (2009). Bile acids as regulatory molecules. *J. Lipid Res.* 50 1509–1520. 10.1194/jlr.R900007-JLR200 19346331 PMC2724047

[B25] Jorgensen W. L. Chandrasekhar J. Madura J. D. Impey R. W. Klein M. L. (1983). Comparison of simple potential functions for simulating liquid water. *J. Chem. Phys.* 79 926–935. 10.1063/1.445869

[B26] Liu H. Mueller-Plathe F. Van Gunsteren W. F. (1995). A force field for liquid dimethyl sulfoxide and physical properties of liquid dimethyl sulfoxide calculated using molecular dynamics simulation. *J. Am. Chem. Soc.* 117 4363–4366. 10.1021/ja00120a018

[B27] Mark A. E. Vanhelden S. P. Smith P. E. Janssen L. H. M. Vangunsteren W. F. (1994). Convergence properties of free energy calculations: alpha-cyclodextrin complexes as a case study. *J. Am. Chem. Soc.* 116 6293–6302. 10.1021/ja00093a032

[B28] Marrink S. J. Berendsen H. J. C. (1994). Simulation of water transport through a lipid membrane. *J. Phys. Chem.* 98 4155–4168. 10.1021/j100066a040

[B29] Marrink S. J. Mark A. E. (2002). Molecular dynamics simulations of mixed micelles modeling human bile. *Biochemistry* 41 5375–5382. 10.1021/bi015613i 11969397

[B30] Mello-Vieira J. Sousa T. Coutinho A. Fedorov A. Lucas S. D. Moreira R. (2013). Cytotoxic bile acids, but not cytoprotective species, inhibit the ordering effect of cholesterol in model membranes at physiologically active concentrations. *Biochim. Biophys. Acta* 1828 2152–2163. 10.1016/j.bbamem.2013.05.021 23747364

[B31] Monte M. J. Marin J. J. G. Antelo A. Vazquez-Tato J. (2009). Bile acids: chemistry, physiology, and pathophysiology. *World J. Gastroenterol.* 15 804–816. 10.3748/wjg.15.804 19230041 PMC2653380

[B32] Moroi Y. Kitagawa M. Itoh H. (1992). Aqueous solubility and acidity constants of cholic, deoxycholic, chenodeoxycholic, and ursodeoxycholic acids. *J. Lipid Res.* 33 49–53. 1552232

[B33] Mustan F. Ivanova A. Madjarova G. Tcholakova S. Denkov N. (2015). Molecular dynamics simulation of the aggregation patterns in aqueous solutions of bile salts at physiological conditions. *J. Phys. Chem. B* 119 15631–15643. 10.1021/acs.jpcb.5b07063 26605858

[B34] Pártay L. B. Jedlovszky P. Sega M. (2007). Molecular aggregates in aqueous solutions of bile acid salts. molecular dynamics simulation study. *J. Phys. Chem. B* 111 9886–9896. 10.1021/jp072974k 17661512

[B35] Pronk S. Páll S. Schulz R. Larsson P. Bjelkmar P. Apostolov R. (2013). GROMACS 4.5: a high-throughput and highly parallel open source molecular simulation toolkit. *Bioinformatics* 29 845–854. 10.1093/bioinformatics/btt055 23407358 PMC3605599

[B36] Roda A. Hofmann A. F. Mysels K. J. (1983). The influence of bile salt structure on self-association in aqueous solutions. *J. Biol. Chem.* 258 6362–6370. 6853487

[B37] Russell D. W. (2003). The enzymes, regulation, and genetics of bile acid synthesis. *Ann. Rev. Biochem.* 72 137–174. 10.1146/annurev.biochem.72.121801.161712 12543708

[B38] Schmidt M. W. Baldridge K. K. Boatz J. A. Elbert S. T. Gordon M. S. Jensen J. H. (1993). General atomic and molecular electronic structure system. *J. Comput. Chem.* 14 1347–1363. 10.1002/jcc.540141112

[B39] Singh U. C. Kollman P. A. (1984). An approach to computing electrostatic charges for molecules. *J. Comput. Chem.* 5 129–145. 10.1002/jcc.540050204

[B40] Urdaneta V. Casadesús J. (2017). Interactions between bacteria and bile salts in the gastrointestinal and hepatobiliary tracts. *Front. Med.* 4:163. 10.3389/fmed.2017.00163 29043249 PMC5632352

[B41] Van Buuren A. R. Berendsen H. J. C. (1993). Molecular dynamics simulation of the stability of a 22-residue α-helix in water and 30% trifluoroethanol. *Biopolymers* 33 1159–1166. 10.1002/bip.360330802 8364151

[B42] Van Buuren A. R. Marrink S. J. Berendsen H. J. C. (1993). A molecular dynamics study of the decane/water interface. *J. Phys. Chem.* 97 9206–9212. 10.1021/j100138a023

[B43] Van Gunsteren W. Berendsen H. J. C. (eds) (1987). *Gromos-87 manual.* Groningen: Biomos BV Nijenborgh.

[B44] Woollett L. A. Wang Y. Buckley D. D. Yao L. Chin S. Granholm N. (2006). Micellar solubilisation of cholesterol is essential for absorption in humans. *Gut* 55 197–204. 10.1136/gut.2005.069906 16407385 PMC1856496

[B45] Yang L. Feng F. Paul Fawcett J. Tucker I. G. (2015). Kinetic and equilibrium studies of bile salt–liposome interactions. *J. Liposome Res.* 25 58–66. 10.3109/08982104.2014.928888 24960448

