# Supplementary material for: Interaction of Bile Salts With Lipid Bilayers: An Atomistic Molecular Dynamics Study
Source: Front Physiol. 2019 Apr 9;10:393. doi: 10.3389/fphys.2019.00393 (PMC6465969; doi:10.3389/fphys.2019.00393)
Supplement: Supplementary file 1 [file Data_Sheet_1.pdf]

INTERACTION OF BILE SALTS WITH LIPID BILAYERS: AN ATOMISTIC MOLECULAR DYNAMICS STUDY

M. C. Neves,[1,2] H. A. L. Filipe,[1,2,3*] Rita Leones Reis,[1,2] J. P. Prates Ramalho,[4,5] Filipe Coreta-Gomes, [2,6] M. J. Moreno,[1,2,3] and L. M. S. Loura [2,3,7*]

1 - Departamento de Química, Faculdade de Ciências e Tecnologia, Universidade de Coimbra, Largo D. Dinis, Rua Larga, P-3004-535 Coimbra, Portugal

2 - Centro de Química de Coimbra, Largo D. Dinis, Rua Larga, P-3004-535 Coimbra, Portugal

3 - Centro de Neurociências e Biologia Celular, Universidade de Coimbra, Rua Larga, P-3004-504 Coimbra, Portugal

4 - Departamento de Química, Escola de Ciências e Tecnologia, Universidade de Évora, Rua Romão Ramalho, 59, P-7000-671 Évora, Portugal

5 - Centro de Química de Évora, Universidade de Évora, Rua Romão Ramalho, 59, P-7000-671 Évora, Portugal

6 - QOPNA & LAQV-REQUIMTE, Departamento de Química, Universidade de Aveiro, P-3810-193 Aveiro, Portugal.

7 - Faculdade de Farmácia, Universidade de Coimbra, Pólo das Ciências da Saúde, Azinhaga de Santa Comba, P-3000-548 Coimbra, Portugal

* Corresponding authors

Telephone: +351 239854481; Fax: +351 239827703; E-mail: hugolourofilipe@gmail.com

Telephone: +351 239488485; Fax: +351 239827126; E-mail: lloura@ff.uc.pt

Contents

GROMACS include topology files for the studied bile salts......................................3
CA...3
DCA...7
CDCA...11
CAH...16
DCAH...20
CDCAH...25
GCA..29
GDCA...34
GCDCA...39

Figure S1 – Final snapshots of the simulations with initial BS location in the water medium...45

Figure S2 – Time evolution of the BS center of mass *z* coordinate for all simulated molecules across the different systems..46

Detailed description of the effect of BS on the POPC headgroup............................47

Figure S3 – Distributions of POPC P-N tilt relative to the bilayer normal, POPC N4 distance relative to the center of the bilayer, and POPC P8 distance relative to the center of the bilayer, for lipid molecules at distance $R < 0.6$ nm to the closest BS molecule ...49

Figure S4 – Distributions of POPC P-N tilt relative to the bilayer normal, POPC N4 distance relative to the center of the bilayer, and POPC P8 distance relative to the center of the bilayer, for lipid molecules at distance 0.6 nm $< R <$ 1.2 nm to the closest BS molecule 50

Table S1 – Average tilt angle of the lipid P-N vector, and average position of N and P atoms, for the lipids that are within 0.6 nm from the BS molecules. 51

GROMACS include topology files for the studied bile salts

CA

```
[ moleculetype ]
;name    nrexcl
CA         3

[ atoms ]
;   nr    type   resnr  residu    atom    cgnr    charge
     1     CH3       1     CA      C1        0    -0.226
     2      CB       1     CA      C2        1     0.089
     3     CH2       1     CA      C3        2     0.016
     4     CH2       1     CA      C4        3    -0.246
     5     CH1       1     CA      C5        4     0.667
     6      OA       1     CA      O6        4    -0.828
     7      HO       1     CA      H7        4     0.452
     8     CH2       1     CA      C8        5    -0.359
     9     CH1       1     CA      C9        6     0.381
    10     CH2       1     CA      C10       7    -0.251
    11     CH1       1     CA      C11       8     0.573
    12      OA       1     CA      O12       8    -0.826
    13      HO       1     CA      H13       8     0.457
    14     CH1       1     CA      C14       9    -0.755
    15     CH1       1     CA      C15      10     0.955
    16     CH2       1     CA      C16      11    -0.433
    17     CH1       1     CA      C17      12     0.321
    18      OA       1     CA      O18      12    -0.806
    19      HO       1     CA      H19      12     0.454
    20      CB       1     CA      C20      13     0.527
    21     CH3       1     CA      C21      14    -0.240
    22     CH1       1     CA      C22      15     0.337
    23     CH2       1     CA      C23      16    -0.105
    24     CH2       1     CA      C24      17    -0.005
    25     CH1       1     CA      C25      18    -0.321
    26     CH1       1     CA      C26      19     0.330
    27     CH3       1     CA      C27      20    -0.206
    28     CH2       1     CA      C28      21     0.054
    29     CH2       1     CA      C29      22    -0.228
    30       C       1     CA      C30      23     1.030
    31      OM       1     CA      O31      23    -0.904
    32      OM       1     CA      O32      23    -0.904

[ bonds ]
;  ai    aj funct         c0           c1
    1     2     1      0.153       334720.
    2     3     1      0.153       334720.
    3     4     1
    4     5     1
    5     6     1
    6     7     1
    5     8     1
    8     9     1
    9     2     1      0.153       334720.
    9    10     1
```

```
   10    11     1        0.153          334720.
   11    14     1
   14    15     1
    2    15     1        0.153          334720.
   15    16     1
   16    17     1
   17    20     1        0.153          334720.
   20    21     1        0.153          334720.
   22    20     1        0.153          334720.
   22    14     1
   22    23     1
   23    24     1
   24    25     1
   20    25     1        0.153          334720.
   25    26     1
   26    27     1
   26    28     1
   28    29     1
   11    12     1
   12    13     1
   17    18     1
   18    19     1
   29    30     1
   30    31     1
   30    32     1

[ pairs ]
;  ai    aj funct            c0            c1
    1     4     1
    1     8     1
    1    10     1
    1    14     1
    1    16     1
    2     5     1
    2    11     1
    2    17     1
    2    22     1
    3     6     1
    3     8     1
    3    10     1
    3    14     1
    3    16     1
    4     7     1
    4     9     1
    4    15     1
    5    10     1
    6     9     1
    7     8     1
    8    11     1
    8    15     1
    9    14     1
    9    16     1
   10    15     1
   10    22     1
   11    16     1
   11    20     1
```

```
  11    23     1
  14    17     1
  14    21     1
  14    24     1
  14    25     1
  15    20     1
  15    23     1
  16    21     1
  16    22     1
  16    25     1
  17    23     1
  17    24     1
  17    26     1
  20    27     1
  20    28     1
  21    23     1
  21    24     1
  21    26     1
  22    26     1
  23    26     1
  24    27     1
  24    28     1
  25    29     1
  26    30     1
  27    29     1
   9    12     1
  15    12     1
  22    12     1
  10    13     1
  14    13     1
  19    16     1
  19    20     1
  18    15     1
  18    22     1
  18    25     1
  18    21     1
  28    31     1
  28    32     1


[ angles ]
;  ai    aj    ak funct          c0          c1
    9     2    15     1          111.        460.240
    9     2     1     1          109.5       460.240
    9     2     3     1          111.        460.240
   15     2     1     1          111.        460.240
   15     2     3     1          111.        460.240
    1     2     3     1          111.        460.240
    2     3     4     1          111.        460.240
    3     4     5     1
    4     5     8     1
    4     5     6     1
    8     5     6     1
    7     6     5     1
    9     8     5     1
    8     9    10     1
```

```
    8     9     2     1      111.          460.240
   10     9     2     1      111.          460.240
   11    10     9     1
   14    11    10     1      111.          460.240
   15    14    22     1
   15    14    11     1
   22    14    11     1
   16    15    14     1
   16    15     2     1      111.          460.240
   14    15     2     1      111.          460.240
   17    16    15     1
   20    17    16     1      111.          460.240
   25    20    21     1      111.          460.240
   25    20    22     1      111.          460.240
   25    20    17     1      111.          460.240
   21    20    22     1      111.          460.240
   21    20    17     1      111.          460.240
   22    20    17     1      111.          460.240
   23    22    20     1      111.          460.240
   23    22    14     1
   20    22    14     1      111.          460.240
   22    23    24     1
   23    24    25     1
   24    25    26     1
   24    25    20     1      111.          460.240
   26    25    20     1      111.          460.240
   27    26    28     1
   27    26    25     1
   28    26    25     1
   29    28    26     1
   10    11    12     1
   14    11    12     1
   11    12    13     1
   16    17    18     1
   20    17    18     1      109.500       460.240
   17    18    19     1
   28    29    30     1
   29    30    31     1
   29    30    32     1
   31    30    32     1

[ dihedrals ]
;  ai    aj    ak    al funct
    4     5     6     7     1     0.000        1.255       3
    4     5     8     9     1     0.000        5.858       3
    8     5     4     3     1     0.000        5.858       3
    5     4     3     2     1     0.000        5.858       3
    4     3     2     9     1     0.000        0.418       6
    9     2    15    16     1     0.000        0.418       6
   15     2     9     8     1     0.000        0.418       6
    2     9    10    11     1     0.000        5.858       3
    2     9     8     5     1     0.000        5.858       3
    9    10    11    14     1     0.000        5.858       3
   10    11    14    15     1     0.000        5.858       3
   15    14    22    23     1     0.000        5.858       3
   22    14    15    16     1     0.000        5.858       3
```

```
   14    15    16    17     1      0.000       5.858      3
   15    16    17    20     1      0.000       5.858      3
   16    17    20    25     1      0.000       0.418      6
   17    20    22    23     1      0.000       0.418      6
   17    20    25    26     1      0.000       0.418      6
   24    25    26    27     1      0.000       5.858      3
   23    24    25    26     1      0.000       5.858      3
   25    24    23    22     1      0.000       5.858      3
   24    23    22    14     1      0.000       5.858      3
   25    26    28    29     1      0.000       5.858      3
   26    28    29    30     1      0.000       5.858      3
   28    29    30    32     1      0.000       0.418      6
   10    11    12    13     1      0.000       1.255      3
   16    17    18    19     1      0.000       1.255      3


[ dihedrals ]
;  ai    aj    ak    al funct
    2    15     3     1     2    35.264     334.720
    5     4     8     6     2    35.264     334.720
   14    15    22    11     2    35.264     334.720
   15    16     2    14     2    35.264     334.720
   20    17    25    22     2    35.264     334.720
   22    14    23    20     2    35.264     334.720
   25    26    20    24     2    35.264     334.720
   26    28    25    27     2    35.264     334.720
    9     8     2    10     2    35.264     334.720
   11    10    14    12     2    35.264     334.720
   17    20    16    18     2    35.264     334.720
   30    32    31    29     2     0.000     167.360
```

DCA

```
[ moleculetype ]
;name    nrexcl
DCA         3

[ atoms ]
;   nr    type   resnr  residu    atom    cgnr    charge
     1     CH3       1    DCA      C1        0    -0.244
     2      CB       1    DCA      C2        1     0.162
     3     CH2       1    DCA      C3        2     0.083
     4     CH2       1    DCA      C4        3    -0.291
     5     CH1       1    DCA      C5        4     0.680
     6      OA       1    DCA      O6        4    -0.830
     7      HO       1    DCA      H7        4     0.451
     8     CH2       1    DCA      C8        5    -0.344
     9     CH1       1    DCA      C9        6     0.254
    10     CH2       1    DCA      C10       7    -0.159
    11     CH2       1    DCA      C11       8     0.061
    12     CH1       1    DCA      C12       9    -0.288
    13     CH1       1    DCA      C13      10     0.686
    14     CH2       1    DCA      C14      11    -0.410
    15     CH1       1    DCA      C15      12     0.300
    16      OA       1    DCA      O16      12    -0.801
```

```
   17      HO      1    DCA     H17     12     0.453
   18      CB      1    DCA     C18     13     0.552
   19     CH3      1    DCA     C19     14    -0.254
   20     CH1      1    DCA     C20     15     0.144
   21     CH2      1    DCA     C21     16    -0.076
   22     CH2      1    DCA     C22     17    -0.026
   23     CH1      1    DCA     C23     18    -0.240
   24     CH1      1    DCA     C24     19     0.300
   25     CH3      1    DCA     C25     20    -0.210
   26     CH2      1    DCA     C26     21     0.042
   27     CH2      1    DCA     C27     22    -0.207
   28       C      1    DCA     C28     23     1.028
   29      OM      1    DCA     O29     23    -0.908
   30      OM      1    DCA     O30     23    -0.908


[ bonds ]
;  ai    aj funct          c0           c1
    1     2     1       0.153         334720.
    2     3     1       0.153         334720.
    3     4     1
    4     5     1
    5     6     1
    6     7     1
    5     8     1
    8     9     1
    9     2     1       0.153         334720.
    9    10     1
   10    11     1       0.153         334720.
   11    12     1
   12    13     1
    2    13     1       0.153         334720.
   13    14     1
   14    15     1
   15    18     1       0.153         334720.
   18    19     1       0.153         334720.
   20    18     1       0.153         334720.
   20    12     1
   20    21     1
   21    22     1
   22    23     1
   18    23     1       0.153         334720.
   23    24     1
   24    25     1
   24    26     1
   26    27     1
   15    16     1
   16    17     1
   27    28     1
   28    29     1
   28    30     1

[ pairs ]
;  ai    aj funct          c0           c1
   1     4     1
   1     8     1
```

1	10	1
1	12	1
1	14	1
2	5	1
2	11	1
2	15	1
2	20	1
3	6	1
3	8	1
3	10	1
3	12	1
3	14	1
4	7	1
4	9	1
4	13	1
5	10	1
6	9	1
7	8	1
8	11	1
8	13	1
9	12	1
9	14	1
10	13	1
10	20	1
11	14	1
11	18	1
11	21	1
12	15	1
12	19	1
12	22	1
12	23	1
13	18	1
13	21	1
14	19	1
14	20	1
14	23	1
15	21	1
15	22	1
15	24	1
18	25	1
18	26	1
19	21	1
19	22	1
19	24	1
20	24	1
21	24	1
22	25	1
22	26	1
23	27	1
24	28	1
25	27	1
17	14	1
17	18	1
16	13	1
16	20	1
16	23	1

```
   16    19     1
   26    29     1
   26    30     1

[ angles ]
;  ai    aj    ak funct            c0            c1
    9     2    13     1            111.           460.240
    9     2     1     1            109.5          460.240
    9     2     3     1            111.           460.240
   13     2     1     1            111.           460.240
   13     2     3     1            111.           460.240
    1     2     3     1            111.           460.240
    2     3     4     1            111.           460.240
    3     4     5     1
    4     5     8     1
    4     5     6     1
    8     5     6     1
    7     6     5     1
    9     8     5     1
    8     9    10     1
    8     9     2     1            111.           460.240
   10     9     2     1            111.           460.240
   11    10     9     1
   12    11    10     1            111.           460.240
   13    12    20     1
   13    12    11     1
   20    12    11     1
   14    13    12     1
   14    13     2     1            111.           460.240
   12    13     2     1            111.           460.240
   15    14    13     1
   18    15    14     1            111.           460.240
   23    18    19     1            111.           460.240
   23    18    20     1            111.           460.240
   23    18    15     1            111.           460.240
   19    18    20     1            111.           460.240
   19    18    15     1            111.           460.240
   20    18    15     1            111.           460.240
   21    20    18     1            111.           460.240
   21    20    12     1
   18    20    12     1            111.           460.240
   20    21    22     1
   21    22    23     1
   22    23    24     1
   22    23    18     1            111.           460.240
   24    23    18     1            111.           460.240
   25    24    26     1
   25    24    23     1
   26    24    23     1
   27    26    24     1
   14    15    16     1
   18    15    16     1            109.500        460.240
   15    16    17     1
   26    27    28     1
   27    28    29     1
```

```
   27    28    30     1
   29    28    30     1

[ dihedrals ]
;  ai    aj    ak    al  funct
    4     5     6     7     1    0.000        1.255       3
    4     5     8     9     1    0.000        5.858       3
    8     5     4     3     1    0.000        5.858       3
    5     4     3     2     1    0.000        5.858       3
    4     3     2     9     1    0.000        0.418       6
    9     2    13    14     1    0.000        0.418       6
   13     2     9     8     1    0.000        0.418       6
    2     9    10    11     1    0.000        5.858       3
    2     9     8     5     1    0.000        5.858       3
    9    10    11    12     1    0.000        5.858       3
   10    11    12    13     1    0.000        5.858       3
   13    12    20    21     1    0.000        5.858       3
   20    12    13    14     1    0.000        5.858       3
   12    13    14    15     1    0.000        5.858       3
   13    14    15    18     1    0.000        5.858       3
   14    15    18    23     1    0.000        0.418       6
   15    18    20    21     1    0.000        0.418       6
   15    18    23    24     1    0.000        0.418       6
   22    23    24    25     1    0.000        5.858       3
   21    22    23    24     1    0.000        5.858       3
   23    22    21    20     1    0.000        5.858       3
   22    21    20    12     1    0.000        5.858       3
   23    24    26    27     1    0.000        5.858       3
   24    26    27    28     1    0.000        5.858       3
   26    27    28    30     1    0.000        0.418       6
   14    15    16    17     1    0.000        1.255       3


[ dihedrals ]
;  ai    aj    ak    al  funct
    2    13     3     1     2    35.264     334.720
    5     4     8     6     2    35.264     334.720
   12    13    20    11     2    35.264     334.720
   13    14     2    12     2    35.264     334.720
   18    15    23    20     2    35.264     334.720
   20    12    21    18     2    35.264     334.720
   23    24    18    22     2    35.264     334.720
   24    26    23    25     2    35.264     334.720
    9     8     2    10     2    35.264     334.720
   15    18    14    16     2    35.264     334.720
   28    30    27    29     2     0.000     167.360
```

CDCA

```
[ moleculetype ]
;name    nrexcl
CDCA         3

[ atoms ]
```

```
;    nr     type   resnr  residu    atom    cgnr     charge
      1      CH3       1   CDCA      C1        0     -0.211
      2       CB       1   CDCA      C2        1      0.072
      3      CH2       1   CDCA      C3        2      0.015
      4      CH2       1   CDCA      C4        3     -0.225
      5      CH1       1   CDCA      C5        4      0.656
      6       OA       1   CDCA      O6        4     -0.824
      7       HO       1   CDCA      H7        4      0.450
      8      CH2       1   CDCA      C8        5     -0.351
      9      CH1       1   CDCA      C9        6      0.409
     10      CH2       1   CDCA      C10       7     -0.260
     11      CH1       1   CDCA      C11       8      0.567
     12       OA       1   CDCA      O12       8     -0.836
     13       HO       1   CDCA      H13       8      0.464
     14      CH1       1   CDCA      C14       9     -0.571
     15      CH1       1   CDCA      C15      10      0.658
     16      CH2       1   CDCA      C16      11     -0.256
     17      CH2       1   CDCA      C17      12     -0.176
     18       CB       1   CDCA      C18      13      0.896
     19      CH3       1   CDCA      C19      14     -0.291
     20      CH1       1   CDCA      C20      15      0.144
     21      CH2       1   CDCA      C21      16     -0.121
     22      CH2       1   CDCA      C22      17      0.010
     23      CH1       1   CDCA      C23      18     -0.379
     24      CH1       1   CDCA      C24      19      0.332
     25      CH3       1   CDCA      C25      20     -0.222
     26      CH2       1   CDCA      C26      21      0.062
     27      CH2       1   CDCA      C27      22     -0.227
     28        C       1   CDCA      C28      26      1.027
     29       OM       1   CDCA      O29      26     -0.906
     30       OM       1   CDCA      O30      26     -0.906


 [ bonds ]
;  ai    aj  funct           c0            c1
    1     2      1        0.153        334720.
    2     3      1        0.153        334720.
    3     4      1
    4     5      1
    5     6      1
    6     7      1
    5     8      1
    8     9      1
    9     2      1        0.153        334720.
    9    10      1
   10    11      1        0.153        334720.
   11    14      1
   14    15      1
    2    15      1        0.153        334720.
   15    16      1
   16    17      1
   17    18      1        0.153        334720.
   18    19      1        0.153        334720.
   20    18      1        0.153        334720.
   20    14      1
   20    21      1
```

```
    21    22     1
    22    23     1
    18    23     1         0.153         334720.
    23    24     1
    24    25     1
    24    26     1
    26    27     1
    11    12     1
    12    13     1
    27    28     1
    28    29     1
    28    30     1

[ pairs ]
;  ai     aj funct            c0            c1
    1      4     1
    1      8     1
    1     10     1
    1     14     1
    1     16     1
    2      5     1
    2     11     1
    2     17     1
    2     20     1
    3      6     1
    3      8     1
    3     10     1
    3     14     1
    3     16     1
    4      7     1
    4      9     1
    4     15     1
    5     10     1
    6      9     1
    7      8     1
    8     11     1
    8     15     1
    9     14     1
    9     16     1
   10     15     1
   10     20     1
   11     16     1
   11     18     1
   11     21     1
   14     17     1
   14     19     1
   14     22     1
   14     23     1
   15     18     1
   15     21     1
   16     19     1
   16     20     1
   16     23     1
   17     21     1
   17     22     1
   17     24     1
```

```
  18    25     1
  18    26     1
  19    21     1
  19    22     1
  19    24     1
  20    24     1
  21    24     1
  22    25     1
  22    26     1
  23    27     1
  24    28     1
  25    27     1
   9    12     1
  15    12     1
  20    12     1
  10    13     1
  14    13     1
  26    29     1
  26    30     1


[ angles ]
;  ai    aj    ak funct            c0            c1
     9     2    15     1           111.          460.240
     9     2     1     1           109.5         460.240
     9     2     3     1           111.          460.240
    15     2     1     1           111.          460.240
    15     2     3     1           111.          460.240
     1     2     3     1           111.          460.240
     2     3     4     1           111.          460.240
     3     4     5     1
     4     5     8     1
     4     5     6     1
     8     5     6     1
     7     6     5     1
     9     8     5     1
     8     9    10     1
     8     9     2     1           111.          460.240
    10     9     2     1           111.          460.240
    11    10     9     1
    14    11    10     1           111.          460.240
    15    14    20     1
    15    14    11     1
    20    14    11     1
    16    15    14     1
    16    15     2     1           111.          460.240
    14    15     2     1           111.          460.240
    17    16    15     1
    18    17    16     1           111.          460.240
    23    18    19     1           111.          460.240
    23    18    20     1           111.          460.240
    23    18    17     1           111.          460.240
    19    18    20     1           111.          460.240
    19    18    17     1           111.          460.240
    20    18    17     1           111.          460.240
    21    20    18     1           111.          460.240
```

```
   21    20    14     1
   18    20    14     1          111.         460.240
   20    21    22     1
   21    22    23     1
   22    23    24     1
   22    23    18     1          111.         460.240
   24    23    18     1          111.         460.240
   25    24    26     1
   25    24    23     1
   26    24    23     1
   27    26    24     1
   10    11    12     1
   14    11    12     1
   11    12    13     1
   26    27    28     1
   27    28    29     1
   27    28    30     1
   29    28    30     1

[ dihedrals ]
;  ai    aj    ak    al funct
    4     5     6     7     1     0.000       1.255      3
    4     5     8     9     1     0.000       5.858      3
    8     5     4     3     1     0.000       5.858      3
    5     4     3     2     1     0.000       5.858      3
    4     3     2     9     1     0.000       0.418      6
    9     2    15    16     1     0.000       0.418      6
   15     2     9     8     1     0.000       0.418      6
    2     9    10    11     1     0.000       5.858      3
    2     9     8     5     1     0.000       5.858      3
    9    10    11    14     1     0.000       5.858      3
   10    11    14    15     1     0.000       5.858      3
   15    14    20    21     1     0.000       5.858      3
   20    14    15    16     1     0.000       5.858      3
   14    15    16    17     1     0.000       5.858      3
   15    16    17    18     1     0.000       5.858      3
   16    17    18    23     1     0.000       0.418      6
   17    18    20    21     1     0.000       0.418      6
   17    18    23    24     1     0.000       0.418      6
   22    23    24    25     1     0.000       5.858      3
   21    22    23    24     1     0.000       5.858      3
   23    22    21    20     1     0.000       5.858      3
   22    21    20    14     1     0.000       5.858      3
   23    24    26    27     1     0.000       5.858      3
   24    26    27    28     1     0.000       5.858      3
   26    27    28    30     1     0.000       0.418      6
   10    11    12    13     1     0.000       1.255      3


[ dihedrals ]
;  ai    aj    ak    al funct
    2    15     3     1     2    35.264     334.720
    5     4     8     6     2    35.264     334.720
   14    15    20    11     2    35.264     334.720
   15    16     2    14     2    35.264     334.720
   18    17    23    20     2    35.264     334.720
```

```
   20    14    21    18     2    35.264     334.720
   23    24    18    22     2    35.264     334.720
   24    26    23    25     2    35.264     334.720
    9     8     2    10     2    35.264     334.720
   11    10    14    12     2    35.264     334.720
   28    30    27    29     2     0.000     167.360
```

CAH

```
[ moleculetype ]
;name    nrexcl
CAH         3

[ atoms ]
;   nr    type   resnr  residu    atom    cgnr    charge
     1     CH3       1     CAH      C1       0    -0.173
     2      CB       1     CAH      C2       1    -0.146
     3     CH2       1     CAH      C3       2     0.079
     4     CH2       1     CAH      C4       3    -0.298
     5     CH1       1     CAH      C5       4     0.738
     6      OA       1     CAH      O6       4    -0.822
     7      HO       1     CAH      H7       4     0.461
     8     CH2       1     CAH      C8       5    -0.507
     9     CH1       1     CAH      C9       6     0.635
    10     CH2       1     CAH     C10       7    -0.432
    11     CH1       1     CAH     C11       8     0.958
    12      OA       1     CAH     O12       8    -0.866
    13      HO       1     CAH     H13       8     0.467
    14     CH1       1     CAH     C14       9    -1.190
    15     CH1       1     CAH     C15      10     1.072
    16     CH2       1     CAH     C16      11    -0.435
    17     CH1       1     CAH     C17      12     0.205
    18      OA       1     CAH     O18      12    -0.807
    19      HO       1     CAH     H19      12     0.477
    20      CB       1     CAH     C20      13     0.662
    21     CH3       1     CAH     C21      14    -0.232
    22     CH1       1     CAH     C22      15     0.485
    23     CH2       1     CAH     C23      16    -0.113
    24     CH2       1     CAH     C24      17    -0.021
    25     CH1       1     CAH     C25      18    -0.299
    26     CH1       1     CAH     C26      19     0.399
    27     CH3       1     CAH     C27      20    -0.195
    28     CH2       1     CAH     C28      21     0.020
    29     CH2       1     CAH     C29      22    -0.194
    30       C       1     CAH     C30      23     0.937
    31      OA       1     CAH     O31      23    -0.709
    32      OM       1     CAH     O32      23    -0.598
    33      HO       1     CAH     H33      23     0.442


 [ bonds ]
;  ai    aj  funct           c0           c1
    1     2     1        0.153          334720.
    2     3     1        0.153          334720.
    3     4     1
```

```
    4     5     1
    5     6     1
    6     7     1
    5     8     1
    8     9     1
    9     2     1        0.153         334720.
    9    10     1
   10    11     1        0.153         334720.
   11    14     1
   14    15     1
    2    15     1        0.153         334720.
   15    16     1
   16    17     1
   17    20     1        0.153         334720.
   20    21     1        0.153         334720.
   22    20     1        0.153         334720.
   22    14     1
   22    23     1
   23    24     1
   24    25     1
   20    25     1        0.153         334720.
   25    26     1
   26    27     1
   26    28     1
   28    29     1
   11    12     1
   12    13     1
   17    18     1
   18    19     1
   29    30     1
   30    31     1
   30    32     1
   31    33     1

[ pairs ]
;  ai    aj funct            c0           c1
   1     4     1
   1     8     1
   1    10     1
   1    14     1
   1    16     1
   2     5     1
   2    11     1
   2    17     1
   2    22     1
   3     6     1
   3     8     1
   3    10     1
   3    14     1
   3    16     1
   4     7     1
   4     9     1
   4    15     1
   5    10     1
   6     9     1
   7     8     1
```

```
    8    11      1
    8    15      1
    9    14      1
    9    16      1
   10    15      1
   10    22      1
   11    16      1
   11    20      1
   11    23      1
   14    17      1
   14    21      1
   14    24      1
   14    25      1
   15    20      1
   15    23      1
   16    21      1
   16    22      1
   16    25      1
   17    23      1
   17    24      1
   17    26      1
   20    27      1
   20    28      1
   21    23      1
   21    24      1
   21    26      1
   22    26      1
   23    26      1
   24    27      1
   24    28      1
   25    29      1
   26    30      1
   27    29      1
    9    12      1
   15    12      1
   22    12      1
   10    13      1
   14    13      1
   19    16      1
   19    20      1
   18    15      1
   18    22      1
   18    25      1
   18    21      1
   28    31      1
   28    32      1
   29    33      1
   32    33      1

[ angles ]
;  ai    aj    ak funct            c0           c1
     9     2    15     1           111.         460.240
     9     2     1     1           109.5        460.240
     9     2     3     1           111.         460.240
    15     2     1     1           111.         460.240
```

```
   15     2     3     1          111.         460.240
    1     2     3     1          111.         460.240
    2     3     4     1          111.         460.240
    3     4     5     1
    4     5     8     1
    4     5     6     1
    8     5     6     1
    7     6     5     1
    9     8     5     1
    8     9    10     1
    8     9     2     1          111.         460.240
   10     9     2     1          111.         460.240
   11    10     9     1
   14    11    10     1          111.         460.240
   15    14    22     1
   15    14    11     1
   22    14    11     1
   16    15    14     1
   16    15     2     1          111.         460.240
   14    15     2     1          111.         460.240
   17    16    15     1
   20    17    16     1          111.         460.240
   25    20    21     1          111.         460.240
   25    20    22     1          111.         460.240
   25    20    17     1          111.         460.240
   21    20    22     1          111.         460.240
   21    20    17     1          111.         460.240
   22    20    17     1          111.         460.240
   23    22    20     1          111.         460.240
   23    22    14     1
   20    22    14     1          111.         460.240
   22    23    24     1
   23    24    25     1
   24    25    26     1
   24    25    20     1          111.         460.240
   26    25    20     1          111.         460.240
   27    26    28     1
   27    26    25     1
   28    26    25     1
   29    28    26     1
   10    11    12     1
   14    11    12     1
   11    12    13     1
   16    17    18     1
   20    17    18     1          109.500      460.240
   17    18    19     1
   28    29    30     1
   29    30    31     1
   29    30    32     1
   31    30    32     1          124.000      502.080
   30    31    33     1

[ dihedrals ]
;  ai    aj    ak    al funct
    4     5     6     7     1     0.000        1.255       3
    4     5     8     9     1     0.000        5.858       3
```

```
    8     5     4     3     1     0.000       5.858      3
    5     4     3     2     1     0.000       5.858      3
    4     3     2     9     1     0.000       0.418      6
    9     2    15    16     1     0.000       0.418      6
   15     2     9     8     1     0.000       0.418      6
    2     9    10    11     1     0.000       5.858      3
    2     9     8     5     1     0.000       5.858      3
    9    10    11    14     1     0.000       5.858      3
   10    11    14    15     1     0.000       5.858      3
   15    14    22    23     1     0.000       5.858      3
   22    14    15    16     1     0.000       5.858      3
   14    15    16    17     1     0.000       5.858      3
   15    16    17    20     1     0.000       5.858      3
   16    17    20    25     1     0.000       0.418      6
   17    20    22    23     1     0.000       0.418      6
   17    20    25    26     1     0.000       0.418      6
   24    25    26    27     1     0.000       5.858      3
   23    24    25    26     1     0.000       5.858      3
   25    24    23    22     1     0.000       5.858      3
   24    23    22    14     1     0.000       5.858      3
   25    26    28    29     1     0.000       5.858      3
   26    28    29    30     1     0.000       5.858      3
   28    29    30    32     1     0.000       0.418      6
   29    30    31    33     1   180.000      16.736      2
   10    11    12    13     1     0.000       1.255      3
   16    17    18    19     1     0.000       1.255      3


[ dihedrals ]
;  ai    aj    ak    al  funct
    2    15     3     1     2    35.264     334.720
    5     4     8     6     2    35.264     334.720
   14    15    22    11     2    35.264     334.720
   15    16     2    14     2    35.264     334.720
   20    17    25    22     2    35.264     334.720
   22    14    23    20     2    35.264     334.720
   25    26    20    24     2    35.264     334.720
   26    28    25    27     2    35.264     334.720
    9     8     2    10     2    35.264     334.720
   11    10    14    12     2    35.264     334.720
   17    20    16    18     2    35.264     334.720
   30    32    31    29     2     0.000     167.360
```

DCAH

```
[ moleculetype ]
;name    nrexcl
DCAH          3

[ atoms ]
;   nr     type   resnr  residu     atom     cgnr     charge
     1      CH3       1    DCAH       C1        0     -0.223
     2       CB       1    DCAH       C2        1      0.188
     3      CH2       1    DCAH       C3        2      0.052
```

```
     4      CH2       1     DCAH      C4         3     -0.260
     5      CH1       1     DCAH      C5         4      0.658
     6       OA       1     DCAH      O6         4     -0.816
     7       HO       1     DCAH      H7         4      0.453
     8      CH2       1     DCAH      C8         5     -0.334
     9      CH1       1     DCAH      C9         6      0.270
    10      CH2       1     DCAH      C10        7     -0.150
    11      CH2       1     DCAH      C11        8      0.068
    12      CH1       1     DCAH      C12        9     -0.219
    13      CH1       1     DCAH      C13       10      0.539
    14      CH2       1     DCAH      C14       11     -0.369
    15      CH1       1     DCAH      C15       12      0.194
    16       OA       1     DCAH      O16       12     -0.803
    17       HO       1     DCAH      H17       12      0.480
    18       CB       1     DCAH      C18       13      0.689
    19      CH3       1     DCAH      C19       14     -0.246
    20      CH1       1     DCAH      C20       15      0.067
    21      CH2       1     DCAH      C21       16     -0.064
    22      CH2       1     DCAH      C22       17     -0.025
    23      CH1       1     DCAH      C23       18     -0.221
    24      CH1       1     DCAH      C24       19      0.355
    25      CH3       1     DCAH      C25       20     -0.183
    26      CH2       1     DCAH      C26       21      0.008
    27      CH2       1     DCAH      C27       22     -0.165
    28        C       1     DCAH      C28       23      0.913
    29       OA       1     DCAH      O29       23     -0.703
    30       OM       1     DCAH      O30       23     -0.593
    31       HO       1     DCAH      H31       23      0.440

[ bonds ]
;  ai    aj  funct           c0           c1
    1     2      1        0.153          334720.
    2     3      1        0.153          334720.
    3     4      1
    4     5      1
    5     6      1
    6     7      1
    5     8      1
    8     9      1
    9     2      1        0.153          334720.
    9    10      1
   10    11      1        0.153          334720.
   11    12      1
   12    13      1
    2    13      1        0.153          334720.
   13    14      1
   14    15      1
   15    18      1        0.153          334720.
   18    19      1        0.153          334720.
   20    18      1        0.153          334720.
   20    12      1
   20    21      1
   21    22      1
   22    23      1
   18    23      1        0.153          334720.
```

```
   23    24     1
   24    25     1
   24    26     1
   26    27     1
   15    16     1
   16    17     1
   27    28     1
   28    29     1
   28    30     1
   29    31     1

[ pairs ]
;  ai    aj funct           c0           c1
    1     4     1
    1     8     1
    1    10     1
    1    12     1
    1    14     1
    2     5     1
    2    11     1
    2    15     1
    2    20     1
    3     6     1
    3     8     1
    3    10     1
    3    12     1
    3    14     1
    4     7     1
    4     9     1
    4    13     1
    5    10     1
    6     9     1
    7     8     1
    8    11     1
    8    13     1
    9    12     1
    9    14     1
   10    13     1
   10    20     1
   11    14     1
   11    18     1
   11    21     1
   12    15     1
   12    19     1
   12    22     1
   12    23     1
   13    18     1
   13    21     1
   14    19     1
   14    20     1
   14    23     1
   15    21     1
   15    22     1
   15    24     1
   18    25     1
   18    26     1
```

```
   19     21      1
   19     22      1
   19     24      1
   20     24      1
   21     24      1
   22     25      1
   22     26      1
   23     27      1
   24     28      1
   25     27      1
   17     14      1
   17     18      1
   16     13      1
   16     20      1
   16     23      1
   16     19      1
   26     29      1
   26     30      1
   27     31      1
   30     31      1

[ angles ]
;  ai    aj    ak funct            c0            c1
     9     2    13     1           111.          460.240
     9     2     1     1           109.5         460.240
     9     2     3     1           111.          460.240
    13     2     1     1           111.          460.240
    13     2     3     1           111.          460.240
     1     2     3     1           111.          460.240
     2     3     4     1           111.          460.240
     3     4     5     1
     4     5     8     1
     4     5     6     1
     8     5     6     1
     7     6     5     1
     9     8     5     1
     8     9    10     1
     8     9     2     1           111.          460.240
    10     9     2     1           111.          460.240
    11    10     9     1
    12    11    10     1           111.          460.240
    13    12    20     1
    13    12    11     1
    20    12    11     1
    14    13    12     1
    14    13     2     1           111.          460.240
    12    13     2     1           111.          460.240
    15    14    13     1
    18    15    14     1           111.          460.240
    23    18    19     1           111.          460.240
    23    18    20     1           111.          460.240
    23    18    15     1           111.          460.240
    19    18    20     1           111.          460.240
    19    18    15     1           111.          460.240
    20    18    15     1           111.          460.240
    21    20    18     1           111.          460.240
```

```
   21    20    12     1
   18    20    12     1           111.          460.240
   20    21    22     1
   21    22    23     1
   22    23    24     1
   22    23    18     1           111.          460.240
   24    23    18     1           111.          460.240
   25    24    26     1
   25    24    23     1
   26    24    23     1
   27    26    24     1
   14    15    16     1
   18    15    16     1           109.500       460.240
   15    16    17     1
   26    27    28     1
   27    28    29     1
   27    28    30     1
   29    28    30     1           124.000       502.080
   28    29    31     1

[ dihedrals ]
;  ai    aj    ak    al  funct
    4     5     6     7     1    0.000       1.255       3
    4     5     8     9     1    0.000       5.858       3
    8     5     4     3     1    0.000       5.858       3
    5     4     3     2     1    0.000       5.858       3
    4     3     2     9     1    0.000       0.418       6
    9     2    13    14     1    0.000       0.418       6
   13     2     9     8     1    0.000       0.418       6
    2     9    10    11     1    0.000       5.858       3
    2     9     8     5     1    0.000       5.858       3
    9    10    11    12     1    0.000       5.858       3
   10    11    12    13     1    0.000       5.858       3
   13    12    20    21     1    0.000       5.858       3
   20    12    13    14     1    0.000       5.858       3
   12    13    14    15     1    0.000       5.858       3
   13    14    15    18     1    0.000       5.858       3
   14    15    18    23     1    0.000       0.418       6
   15    18    20    21     1    0.000       0.418       6
   15    18    23    24     1    0.000       0.418       6
   22    23    24    25     1    0.000       5.858       3
   21    22    23    24     1    0.000       5.858       3
   23    22    21    20     1    0.000       5.858       3
   22    21    20    12     1    0.000       5.858       3
   23    24    26    27     1    0.000       5.858       3
   24    26    27    28     1    0.000       5.858       3
   26    27    28    30     1    0.000       0.418       6
   27    28    29    31     1  180.000      16.736        2
   14    15    16    17     1    0.000       1.255       3


[ dihedrals ]
;  ai    aj    ak    al  funct
    2    13     3     1     2    35.264     334.720
    5     4     8     6     2    35.264     334.720
   12    13    20    11     2    35.264     334.720
```

```
   13    14     2    12     2    35.264     334.720
   18    15    23    20     2    35.264     334.720
   20    12    21    18     2    35.264     334.720
   23    24    18    22     2    35.264     334.720
   24    26    23    25     2    35.264     334.720
    9     8     2    10     2    35.264     334.720
   15    18    14    16     2    35.264     334.720
   28    30    27    29     2     0.000     167.360
```

CDCAH

```
[ moleculetype ]
;name    nrexcl
CDCAH         3

[ atoms ]
;   nr    type   resnr  residu    atom    cgnr    charge
     1     CH3       1   CDCAH     C1        0    -0.159
     2      CB       1   CDCAH     C2        1    -0.176
     3     CH2       1   CDCAH     C3        2     0.107
     4     CH2       1   CDCAH     C4        3    -0.285
     5     CH1       1   CDCAH     C5        4     0.711
     6      OA       1   CDCAH     O6        4    -0.817
     7      HO       1   CDCAH     H7        4     0.457
     8     CH2       1   CDCAH     C8        5    -0.450
     9     CH1       1   CDCAH     C9        6     0.576
    10     CH2       1   CDCAH     C10       7    -0.390
    11     CH1       1   CDCAH     C11       8     0.835
    12      OA       1   CDCAH     O12       8    -0.848
    13      HO       1   CDCAH     H13       8     0.458
    14     CH1       1   CDCAH     C14       9    -0.981
    15     CH1       1   CDCAH     C15      10     0.852
    16     CH2       1   CDCAH     C16      11    -0.262
    17     CH2       1   CDCAH     C17      12    -0.172
    18      CB       1   CDCAH     C18      13     0.721
    19     CH3       1   CDCAH     C19      14    -0.261
    20     CH1       1   CDCAH     C20      15     0.479
    21     CH2       1   CDCAH     C21      16    -0.146
    22     CH2       1   CDCAH     C22      17    -0.007
    23     CH1       1   CDCAH     C23      18    -0.345
    24     CH1       1   CDCAH     C24      19     0.409
    25     CH3       1   CDCAH     C25      20    -0.214
    26     CH2       1   CDCAH     C26      21     0.049
    27     CH2       1   CDCAH     C27      22    -0.224
    28       C       1   CDCAH     C28      23     0.952
    29      OA       1   CDCAH     O29      23    -0.713
    30      OM       1   CDCAH     O30      23    -0.602
    31      HO       1   CDCAH     H31      23     0.446


 [ bonds ]
;  ai    aj  funct           c0            c1
    1     2     1        0.153          334720.
    2     3     1        0.153          334720.
```

```
    3     4     1
    4     5     1
    5     6     1
    6     7     1
    5     8     1
    8     9     1
    9     2     1         0.153          334720.
    9    10     1
   10    11     1         0.153          334720.
   11    14     1
   14    15     1
    2    15     1         0.153          334720.
   15    16     1
   16    17     1
   17    18     1         0.153          334720.
   18    19     1         0.153          334720.
   20    18     1         0.153          334720.
   20    14     1
   20    21     1
   21    22     1
   22    23     1
   18    23     1         0.153          334720.
   23    24     1
   24    25     1
   24    26     1
   26    27     1
   11    12     1
   12    13     1
   27    28     1
   28    29     1
   28    30     1
   29    31     1

[ pairs ]
;  ai    aj funct            c0            c1
   1     4     1
   1     8     1
   1    10     1
   1    14     1
   1    16     1
   2     5     1
   2    11     1
   2    17     1
   2    20     1
   3     6     1
   3     8     1
   3    10     1
   3    14     1
   3    16     1
   4     7     1
   4     9     1
   4    15     1
   5    10     1
   6     9     1
   7     8     1
   8    11     1
```

```
   8    15      1
   9    14      1
   9    16      1
  10    15      1
  10    20      1
  11    16      1
  11    18      1
  11    21      1
  14    17      1
  14    19      1
  14    22      1
  14    23      1
  15    18      1
  15    21      1
  16    19      1
  16    20      1
  16    23      1
  17    21      1
  17    22      1
  17    24      1
  18    25      1
  18    26      1
  19    21      1
  19    22      1
  19    24      1
  20    24      1
  21    24      1
  22    25      1
  22    26      1
  23    27      1
  24    28      1
  25    27      1
   9    12      1
  15    12      1
  20    12      1
  10    13      1
  14    13      1
  26    29      1
  26    30      1
  27    31      1
  30    31      1

[ angles ]
;  ai    aj    ak funct            c0           c1
    9     2    15     1            111.         460.240
    9     2     1     1            109.5        460.240
    9     2     3     1            111.         460.240
   15     2     1     1            111.         460.240
   15     2     3     1            111.         460.240
    1     2     3     1            111.         460.240
    2     3     4     1            111.         460.240
    3     4     5     1
    4     5     8     1
    4     5     6     1
    8     5     6     1
    7     6     5     1
```

```
    9     8     5     1
    8     9    10     1
    8     9     2     1          111.          460.240
   10     9     2     1          111.          460.240
   11    10     9     1
   14    11    10     1          111.          460.240
   15    14    20     1
   15    14    11     1
   20    14    11     1
   16    15    14     1
   16    15     2     1          111.          460.240
   14    15     2     1          111.          460.240
   17    16    15     1
   18    17    16     1          111.          460.240
   23    18    19     1          111.          460.240
   23    18    20     1          111.          460.240
   23    18    17     1          111.          460.240
   19    18    20     1          111.          460.240
   19    18    17     1          111.          460.240
   20    18    17     1          111.          460.240
   21    20    18     1          111.          460.240
   21    20    14     1
   18    20    14     1          111.          460.240
   20    21    22     1
   21    22    23     1
   22    23    24     1
   22    23    18     1          111.          460.240
   24    23    18     1          111.          460.240
   25    24    26     1
   25    24    23     1
   26    24    23     1
   27    26    24     1
   10    11    12     1
   14    11    12     1
   11    12    13     1
   26    27    28     1
   27    28    29     1
   27    28    30     1
   29    28    30     1          124.000       502.080
   28    29    31     1

[ dihedrals ]
;  ai    aj    ak    al funct
    4     5     6     7     1     0.000       1.255       3
    4     5     8     9     1     0.000       5.858       3
    8     5     4     3     1     0.000       5.858       3
    5     4     3     2     1     0.000       5.858       3
    4     3     2     9     1     0.000       0.418       6
    9     2    15    16     1     0.000       0.418       6
   15     2     9     8     1     0.000       0.418       6
    2     9    10    11     1     0.000       5.858       3
    2     9     8     5     1     0.000       5.858       3
    9    10    11    14     1     0.000       5.858       3
   10    11    14    15     1     0.000       5.858       3
   15    14    20    21     1     0.000       5.858       3
   20    14    15    16     1     0.000       5.858       3
```

```
   14    15    16    17     1     0.000       5.858      3
   15    16    17    18     1     0.000       5.858      3
   16    17    18    23     1     0.000       0.418      6
   17    18    20    21     1     0.000       0.418      6
   17    18    23    24     1     0.000       0.418      6
   22    23    24    25     1     0.000       5.858      3
   21    22    23    24     1     0.000       5.858      3
   23    22    21    20     1     0.000       5.858      3
   22    21    20    14     1     0.000       5.858      3
   23    24    26    27     1     0.000       5.858      3
   24    26    27    28     1     0.000       5.858      3
   26    27    28    30     1     0.000       0.418      6
   27    28    29    31     1   180.000      16.736      2
   10    11    12    13     1     0.000       1.255      3


[ dihedrals ]
;  ai    aj    ak    al funct
    2    15     3     1     2    35.264     334.720
    5     4     8     6     2    35.264     334.720
   14    15    20    11     2    35.264     334.720
   15    16     2    14     2    35.264     334.720
   18    17    23    20     2    35.264     334.720
   20    14    21    18     2    35.264     334.720
   23    24    18    22     2    35.264     334.720
   24    26    23    25     2    35.264     334.720
    9     8     2    10     2    35.264     334.720
   11    10    14    12     2    35.264     334.720
   28    30    27    29     2     0.000     167.360
```

GCA

```
[ moleculetype ]
;name    nrexcl
GCA         3

[ atoms ]
;   nr    type   resnr  residu    atom    cgnr    charge
     1     CH3       1     GCA     C1        0    -0.208
     2      CB       1     GCA     C2        1     0.121
     3     CH2       1     GCA     C3        2    -0.008
     4     CH2       1     GCA     C4        3    -0.226
     5     CH1       1     GCA     C5        4     0.636
     6      OA       1     GCA     O6        4    -0.816
     7      HO       1     GCA     H7        4     0.455
     8     CH2       1     GCA     C8        5    -0.352
     9     CH1       1     GCA     C9        6     0.413
    10     CH2       1     GCA     C10       7    -0.245
    11     CH1       1     GCA     C11       8     0.580
    12      OA       1     GCA     O12       8    -0.818
    13      HO       1     GCA     H13       8     0.437
    14     CH1       1     GCA     C14       9    -0.694
    15     CH1       1     GCA     C15      10     0.748
    16     CH2       1     GCA     C16      11    -0.419
```

```
    17      CH1        1      GCA      C17       12      0.308
    18       OA        1      GCA      O18       12     -0.798
    19       HO        1      GCA      H19       12      0.465
    20       CB        1      GCA      C20       13      0.523
    21      CH3        1      GCA      C21       14     -0.224
    22      CH1        1      GCA      C22       15      0.451
    23      CH2        1      GCA      C23       16     -0.148
    24      CH2        1      GCA      C24       17      0.019
    25      CH1        1      GCA      C25       18     -0.413
    26      CH1        1      GCA      C26       19      0.418
    27      CH3        1      GCA      C27       20     -0.191
    28      CH2        1      GCA      C28       21      0.178
    29      CH2        1      GCA      C29       22     -0.427
    30        C        1      GCA      C30       23      0.983
    31        O        1      GCA      O31       23     -0.739
    32        N        1      GCA      N32       24     -0.584
    33        H        1      GCA      H33       24      0.325
    34      CH2        1      GCA      C34       25     -0.012
    35        C        1      GCA      C35       26      1.037
    36       OM        1      GCA      O36       26     -0.880
    37       OM        1      GCA      O37       26     -0.895

[ bonds ]
;  ai    aj funct           c0            c1
    1     2     1        0.153         334720.
    2     3     1        0.153         334720.
    3     4     1
    4     5     1
    5     6     1
    6     7     1
    5     8     1
    8     9     1
    9     2     1        0.153         334720.
    9    10     1
   10    11     1        0.153         334720.
   11    14     1
   14    15     1
    2    15     1        0.153         334720.
   15    16     1
   16    17     1
   17    20     1        0.153         334720.
   20    21     1        0.153         334720.
   22    20     1        0.153         334720.
   22    14     1
   22    23     1
   23    24     1
   24    25     1
   20    25     1        0.153         334720.
   25    26     1
   26    27     1
   26    28     1
   28    29     1
   11    12     1
   12    13     1
   17    18     1
```

```
   18    19     1
   29    30     1
   30    31     1
   30    32     1
   32    33     1
   32    34     1
   34    35     1
   35    36     1
   35    37     1

[ pairs ]
;  ai    aj funct            c0            c1
    1     4     1
    1     8     1
    1    10     1
    1    14     1
    1    16     1
    2     5     1
    2    11     1
    2    17     1
    2    22     1
    3     6     1
    3     8     1
    3    10     1
    3    14     1
    3    16     1
    4     7     1
    4     9     1
    4    15     1
    5    10     1
    6     9     1
    7     8     1
    8    11     1
    8    15     1
    9    14     1
    9    16     1
   10    15     1
   10    22     1
   11    16     1
   11    20     1
   11    23     1
   14    17     1
   14    21     1
   14    24     1
   14    25     1
   15    20     1
   15    23     1
   16    21     1
   16    22     1
   16    25     1
   17    23     1
   17    24     1
   17    26     1
   20    27     1
   20    28     1
   21    23     1
```

```
  21    24     1
  21    26     1
  22    26     1
  23    26     1
  24    27     1
  24    28     1
  25    29     1
  26    30     1
  27    29     1
   9    12     1
  15    12     1
  22    12     1
  10    13     1
  14    13     1
  19    16     1
  19    20     1
  18    15     1
  18    22     1
  18    25     1
  18    21     1
  28    31     1
  28    32     1
  29    33     1
  29    34     1
  30    35     1
  31    33     1
  31    34     1
  32    36     1
  32    37     1
  33    35     1


[ angles ]
;  ai    aj    ak funct            c0           c1
     9     2    15     1            111.         460.240
     9     2     1     1            109.5        460.240
     9     2     3     1            111.         460.240
    15     2     1     1            111.         460.240
    15     2     3     1            111.         460.240
     1     2     3     1            111.         460.240
     2     3     4     1            111.         460.240
     3     4     5     1
     4     5     8     1
     4     5     6     1
     8     5     6     1
     7     6     5     1
     9     8     5     1
     8     9    10     1
     8     9     2     1            111.         460.240
    10     9     2     1            111.         460.240
    11    10     9     1
    14    11    10     1            111.         460.240
    15    14    22     1
    15    14    11     1
    22    14    11     1
    16    15    14     1
```

```
   16    15     2     1           111.         460.240
   14    15     2     1           111.         460.240
   17    16    15     1
   20    17    16     1           111.         460.240
   25    20    21     1           111.         460.240
   25    20    22     1           111.         460.240
   25    20    17     1           111.         460.240
   21    20    22     1           111.         460.240
   21    20    17     1           111.         460.240
   22    20    17     1           111.         460.240
   23    22    20     1           111.         460.240
   23    22    14     1
   20    22    14     1           111.         460.240
   22    23    24     1
   23    24    25     1
   24    25    26     1
   24    25    20     1           111.         460.240
   26    25    20     1           111.         460.240
   27    26    28     1
   27    26    25     1
   28    26    25     1
   29    28    26     1
   10    11    12     1
   14    11    12     1
   11    12    13     1
   16    17    18     1
   20    17    18     1           109.500      460.240
   17    18    19     1
   28    29    30     1
   29    30    31     1
   29    30    32     1
   31    30    32     1
   30    32    33     1
   30    32    34     1
   33    32    34     1
   32    34    35     1
   34    35    36     1
   34    35    37     1
   36    35    37     1

[ dihedrals ]
;  ai    aj    ak    al funct
    4     5     6     7     1     0.000       1.255       3
    4     5     8     9     1     0.000       5.858       3
    8     5     4     3     1     0.000       5.858       3
    5     4     3     2     1     0.000       5.858       3
    4     3     2     9     1     0.000       0.418       6
    9     2    15    16     1     0.000       0.418       6
   15     2     9     8     1     0.000       0.418       6
    2     9    10    11     1     0.000       5.858       3
    2     9     8     5     1     0.000       5.858       3
    9    10    11    14     1     0.000       5.858       3
   10    11    14    15     1     0.000       5.858       3
   15    14    22    23     1     0.000       5.858       3
   22    14    15    16     1     0.000       5.858       3
   14    15    16    17     1     0.000       5.858       3
```

```
   15    16    17    20     1      0.000        5.858       3
   16    17    20    25     1      0.000        0.418       6
   17    20    22    23     1      0.000        0.418       6
   17    20    25    26     1      0.000        0.418       6
   24    25    26    27     1      0.000        5.858       3
   23    24    25    26     1      0.000        5.858       3
   25    24    23    22     1      0.000        5.858       3
   24    23    22    14     1      0.000        5.858       3
   25    26    28    29     1      0.000        5.858       3
   26    28    29    30     1      0.000        5.858       3
   28    29    30    32     1      0.000        0.418       6
   10    11    12    13     1      0.000        1.255       3
   16    17    18    19     1      0.000        1.255       3
   29    30    32    34     1    180.000       33.472       2
   30    32    34    35     1    180.000        0.418       6
   32    34    35    36     1      0.000        0.418       6

[ dihedrals ]
;  ai    aj    ak    al funct
    2    15     3     1     2     35.264      334.720
    5     4     8     6     2     35.264      334.720
   14    15    22    11     2     35.264      334.720
   15    16     2    14     2     35.264      334.720
   20    17    25    22     2     35.264      334.720
   22    14    23    20     2     35.264      334.720
   25    26    20    24     2     35.264      334.720
   26    28    25    27     2     35.264      334.720
    9     8     2    10     2     35.264      334.720
   11    10    14    12     2     35.264      334.720
   17    20    16    18     2     35.264      334.720
   30    32    29    31     2      0.000      167.360
   35    37    36    34     2      0.000      167.360
   32    34    33    30     2      0.000      167.360
```

GDCA

```
[ moleculetype ]
;name    nrexcl
GDCA         3

[ atoms ]
;   nr     type   resnr  residu    atom     cgnr    charge
     1      CH3       1    GDCA      C1        0    -0.225
     2       CB       1    GDCA      C2        1     0.152
     3      CH2       1    GDCA      C3        2     0.047
     4      CH2       1    GDCA      C4        3    -0.256
     5      CH1       1    GDCA      C5        4     0.661
     6       OA       1    GDCA      O6        4    -0.824
     7       HO       1    GDCA      H7        4     0.455
     8      CH2       1    GDCA      C8        5    -0.332
     9      CH1       1    GDCA      C9        6     0.273
    10      CH2       1    GDCA     C10        7    -0.154
    11      CH2       1    GDCA     C11        8     0.049
```

```
    12      CH1        1     GDCA      C12        9    -0.182
    13      CH1        1     GDCA      C13       10     0.553
    14      CH2        1     GDCA      C14       11    -0.396
    15      CH1        1     GDCA      C15       12     0.184
    16       OA        1     GDCA      O16       12    -0.800
    17       HO        1     GDCA      H17       12     0.474
    18       CB        1     GDCA      C18       13     0.850
    19      CH3        1     GDCA      C19       14    -0.273
    20      CH1        1     GDCA      C20       15     0.038
    21      CH2        1     GDCA      C21       16    -0.124
    22      CH2        1     GDCA      C22       17     0.031
    23      CH1        1     GDCA      C23       18    -0.339
    24      CH1        1     GDCA      C24       19     0.336
    25      CH3        1     GDCA      C25       20    -0.195
    26      CH2        1     GDCA      C26       21     0.190
    27      CH2        1     GDCA      C27       22    -0.407
    28        C        1     GDCA      C28       23     0.945
    29        O        1     GDCA      O29       23    -0.733
    30        N        1     GDCA      N30       24    -0.583
    31        H        1     GDCA      H31       24     0.321
    32      CH2        1     GDCA      C32       25     0.011
    33        C        1     GDCA      C33       26     1.026
    34       OM        1     GDCA      O34       26    -0.879
    35       OM        1     GDCA      O35       26    -0.895

[ bonds ]
;  ai    aj  funct           c0           c1
    1     2      1       0.153          334720.
    2     3      1       0.153          334720.
    3     4      1
    4     5      1
    5     6      1
    6     7      1
    5     8      1
    8     9      1
    9     2      1       0.153          334720.
    9    10      1
   10    11      1       0.153          334720.
   11    12      1
   12    13      1
    2    13      1       0.153          334720.
   13    14      1
   14    15      1
   15    18      1       0.153          334720.
   18    19      1       0.153          334720.
   20    18      1       0.153          334720.
   20    12      1
   20    21      1
   21    22      1
   22    23      1
   18    23      1       0.153          334720.
   23    24      1
   24    25      1
   24    26      1
   26    27      1
```

```
   15    16     1
   16    17     1
   27    28     1
   28    29     1
   28    30     1
   30    31     1
   30    32     1
   32    33     1
   33    34     1
   33    35     1

[ pairs ]
;  ai    aj funct            c0            c1
    1     4     1
    1     8     1
    1    10     1
    1    12     1
    1    14     1
    2     5     1
    2    11     1
    2    15     1
    2    20     1
    3     6     1
    3     8     1
    3    10     1
    3    12     1
    3    14     1
    4     7     1
    4     9     1
    4    13     1
    5    10     1
    6     9     1
    7     8     1
    8    11     1
    8    13     1
    9    12     1
    9    14     1
   10    13     1
   10    20     1
   11    14     1
   11    18     1
   11    21     1
   12    15     1
   12    19     1
   12    22     1
   12    23     1
   13    18     1
   13    21     1
   14    19     1
   14    20     1
   14    23     1
   15    21     1
   15    22     1
   15    24     1
   18    25     1
   18    26     1
```

```
   19    21      1
   19    22      1
   19    24      1
   20    24      1
   21    24      1
   22    25      1
   22    26      1
   23    27      1
   24    28      1
   25    27      1
   17    14      1
   17    18      1
   16    13      1
   16    20      1
   16    23      1
   16    19      1
   26    29      1
   26    30      1
   27    31      1
   27    32      1
   28    33      1
   29    31      1
   29    32      1
   30    34      1
   30    35      1
   31    33      1

[ angles ]
;  ai    aj    ak funct            c0           c1
    9     2    13     1            111.         460.240
    9     2     1     1            109.5        460.240
    9     2     3     1            111.         460.240
   13     2     1     1            111.         460.240
   13     2     3     1            111.         460.240
    1     2     3     1            111.         460.240
    2     3     4     1            111.         460.240
    3     4     5     1
    4     5     8     1
    4     5     6     1
    8     5     6     1
    7     6     5     1
    9     8     5     1
    8     9    10     1
    8     9     2     1            111.         460.240
   10     9     2     1            111.         460.240
   11    10     9     1
   12    11    10     1            111.         460.240
   13    12    20     1
   13    12    11     1
   20    12    11     1
   14    13    12     1
   14    13     2     1            111.         460.240
   12    13     2     1            111.         460.240
   15    14    13     1
   18    15    14     1            111.         460.240
```

```
   23    18    19     1           111.         460.240
   23    18    20     1           111.         460.240
   23    18    15     1           111.         460.240
   19    18    20     1           111.         460.240
   19    18    15     1           111.         460.240
   20    18    15     1           111.         460.240
   21    20    18     1           111.         460.240
   21    20    12     1
   18    20    12     1           111.         460.240
   20    21    22     1
   21    22    23     1
   22    23    24     1
   22    23    18     1           111.         460.240
   24    23    18     1           111.         460.240
   25    24    26     1
   25    24    23     1
   26    24    23     1
   27    26    24     1
   14    15    16     1
   18    15    16     1           109.500      460.240
   15    16    17     1
   26    27    28     1
   27    28    29     1
   27    28    30     1
   29    28    30     1
   28    30    31     1
   28    30    32     1
   31    30    32     1
   30    32    33     1
   32    33    34     1
   32    33    35     1
   34    33    35     1

[ dihedrals ]
;  ai    aj    ak    al  funct
    4     5     6     7     1    0.000       1.255      3
    4     5     8     9     1    0.000       5.858      3
    8     5     4     3     1    0.000       5.858      3
    5     4     3     2     1    0.000       5.858      3
    4     3     2     9     1    0.000       0.418      6
    9     2    13    14     1    0.000       0.418      6
   13     2     9     8     1    0.000       0.418      6
    2     9    10    11     1    0.000       5.858      3
    2     9     8     5     1    0.000       5.858      3
    9    10    11    12     1    0.000       5.858      3
   10    11    12    13     1    0.000       5.858      3
   13    12    20    21     1    0.000       5.858      3
   20    12    13    14     1    0.000       5.858      3
   12    13    14    15     1    0.000       5.858      3
   13    14    15    18     1    0.000       5.858      3
   14    15    18    23     1    0.000       0.418      6
   15    18    20    21     1    0.000       0.418      6
   15    18    23    24     1    0.000       0.418      6
   22    23    24    25     1    0.000       5.858      3
   21    22    23    24     1    0.000       5.858      3
   23    22    21    20     1    0.000       5.858      3
```

```
   22    21    20    12     1    0.000       5.858      3
   23    24    26    27     1    0.000       5.858      3
   24    26    27    28     1    0.000       5.858      3
   26    27    28    30     1    0.000       0.418      6
   14    15    16    17     1    0.000       1.255      3
   27    28    30    32     1  180.000      33.472      2
   28    30    32    33     1  180.000       0.418      6
   30    32    33    34     1    0.000       0.418      6


[ dihedrals ]
;  ai    aj    ak    al  funct
    2    13     3     1     2    35.264     334.720
    5     4     8     6     2    35.264     334.720
   12    13    20    11     2    35.264     334.720
   13    14     2    12     2    35.264     334.720
   18    15    23    20     2    35.264     334.720
   20    12    21    18     2    35.264     334.720
   23    24    18    22     2    35.264     334.720
   24    26    23    25     2    35.264     334.720
    9     8     2    10     2    35.264     334.720
   15    18    14    16     2    35.264     334.720
   28    30    27    29     2     0.000     167.360
   33    35    34    32     2     0.000     167.360
   30    32    31    28     2     0.000     167.360
```

GCDCA

```
[ moleculetype ]
;name    nrexcl
GCDCA         3

[ atoms ]
;   nr     type   resnr  residu    atom    cgnr    charge
     1      CH3       1   GCDCA      C1       0    -0.196
     2       CB       1   GCDCA      C2       1     0.082
     3      CH2       1   GCDCA      C3       2     0.015
     4      CH2       1   GCDCA      C4       3    -0.228
     5      CH1       1   GCDCA      C5       4     0.642
     6       OA       1   GCDCA      O6       4    -0.816
     7       HO       1   GCDCA      H7       4     0.455
     8      CH2       1   GCDCA      C8       5    -0.359
     9      CH1       1   GCDCA      C9       6     0.435
    10      CH2       1   GCDCA     C10       7    -0.262
    11      CH1       1   GCDCA     C11       8     0.578
    12       OA       1   GCDCA     O12       8    -0.829
    13       HO       1   GCDCA     H13       8     0.456
    14      CH1       1   GCDCA     C14       9    -0.612
    15      CH1       1   GCDCA     C15      10     0.593
    16      CH2       1   GCDCA     C16      11    -0.213
    17      CH2       1   GCDCA     C17      12    -0.149
    18       CB       1   GCDCA     C18      13     0.690
    19      CH3       1   GCDCA     C19      14    -0.243
    20      CH1       1   GCDCA     C20      15     0.286
```

```
    21      CH2        1   GCDCA      C21       16    -0.129
    22      CH2        1   GCDCA      C22       17     0.006
    23      CH1        1   GCDCA      C23       18    -0.377
    24      CH1        1   GCDCA      C24       19     0.362
    25      CH3        1   GCDCA      C25       20    -0.199
    26      CH2        1   GCDCA      C26       21     0.209
    27      CH2        1   GCDCA      C27       22    -0.427
    28        C        1   GCDCA      C28       23     0.980
    29        O        1   GCDCA      O29       23    -0.743
    30        N        1   GCDCA      N30       24    -0.606
    31        H        1   GCDCA      H31       24     0.333
    32      CH2        1   GCDCA      C32       25     0.013
    33        C        1   GCDCA      C33       26     1.021
    34       OM        1   GCDCA      O34       26    -0.879
    35       OM        1   GCDCA      O35       26    -0.892


[ bonds ]
;  ai    aj funct           c0           c1
    1     2     1        0.153        334720.
    2     3     1        0.153        334720.
    3     4     1
    4     5     1
    5     6     1
    6     7     1
    5     8     1
    8     9     1
    9     2     1        0.153        334720.
    9    10     1
   10    11     1        0.153        334720.
   11    14     1
   14    15     1
    2    15     1        0.153        334720.
   15    16     1
   16    17     1
   17    18     1        0.153        334720.
   18    19     1        0.153        334720.
   20    18     1        0.153        334720.
   20    14     1
   20    21     1
   21    22     1
   22    23     1
   18    23     1        0.153        334720.
   23    24     1
   24    25     1
   24    26     1
   26    27     1
   11    12     1
   12    13     1
   27    28     1
   28    29     1
   28    30     1
   30    31     1
   30    32     1
   32    33     1
   33    34     1
```

```
   33    35     1

[ pairs ]
;  ai    aj funct           c0           c1
    1     4     1
    1     8     1
    1    10     1
    1    14     1
    1    16     1
    2     5     1
    2    11     1
    2    17     1
    2    20     1
    3     6     1
    3     8     1
    3    10     1
    3    14     1
    3    16     1
    4     7     1
    4     9     1
    4    15     1
    5    10     1
    6     9     1
    7     8     1
    8    11     1
    8    15     1
    9    14     1
    9    16     1
   10    15     1
   10    20     1
   11    16     1
   11    18     1
   11    21     1
   14    17     1
   14    19     1
   14    22     1
   14    23     1
   15    18     1
   15    21     1
   16    19     1
   16    20     1
   16    23     1
   17    21     1
   17    22     1
   17    24     1
   18    25     1
   18    26     1
   19    21     1
   19    22     1
   19    24     1
   20    24     1
   21    24     1
   22    25     1
   22    26     1
   23    27     1
   24    28     1
```

```
  25    27     1
   9    12     1
  15    12     1
  20    12     1
  10    13     1
  14    13     1
  26    29     1
  26    30     1
  27    31     1
  27    32     1
  28    33     1
  29    31     1
  29    32     1
  30    34     1
  30    35     1
  31    33     1


[ angles ]
;  ai    aj    ak funct            c0           c1
     9     2    15     1            111.         460.240
     9     2     1     1            109.5        460.240
     9     2     3     1            111.         460.240
    15     2     1     1            111.         460.240
    15     2     3     1            111.         460.240
     1     2     3     1            111.         460.240
     2     3     4     1            111.         460.240
     3     4     5     1
     4     5     8     1
     4     5     6     1
     8     5     6     1
     7     6     5     1
     9     8     5     1
     8     9    10     1
     8     9     2     1            111.         460.240
    10     9     2     1            111.         460.240
    11    10     9     1
    14    11    10     1            111.         460.240
    15    14    20     1
    15    14    11     1
    20    14    11     1
    16    15    14     1
    16    15     2     1            111.         460.240
    14    15     2     1            111.         460.240
    17    16    15     1
    18    17    16     1       111.         460.240
    23    18    19     1       111.         460.240
    23    18    20     1       111.         460.240
    23    18    17     1       111.         460.240
    19    18    20     1       111.         460.240
    19    18    17     1       111.         460.240
    20    18    17     1       111.         460.240
    21    20    18     1       111.         460.240
    21    20    14     1
    18    20    14     1       111.         460.240
    20    21    22     1
```

```
   21    22    23     1
   22    23    24     1
   22    23    18     1        111.         460.240
   24    23    18     1        111.         460.240
   25    24    26     1
   25    24    23     1
   26    24    23     1
   27    26    24     1
   10    11    12     1
   14    11    12     1
   11    12    13     1
   26    27    28     1
   27    28    29     1
   27    28    30     1
   29    28    30     1
   28    30    31     1
   28    30    32     1
   31    30    32     1
   30    32    33     1
   32    33    34     1
   32    33    35     1
   34    33    35     1

[ dihedrals ]
;  ai    aj    ak    al funct
    4     5     6     7     1      0.000        1.255       3
    4     5     8     9     1      0.000        5.858       3
    8     5     4     3     1      0.000        5.858       3
    5     4     3     2     1      0.000        5.858       3
    4     3     2     9     1      0.000        0.418       6
    9     2    15    16     1      0.000        0.418       6
   15     2     9     8     1      0.000        0.418       6
    2     9    10    11     1      0.000        5.858       3
    2     9     8     5     1      0.000        5.858       3
    9    10    11    14     1      0.000        5.858       3
   10    11    14    15     1      0.000        5.858       3
   15    14    20    21     1      0.000        5.858       3
   20    14    15    16     1      0.000        5.858       3
   14    15    16    17     1      0.000        5.858       3
   15    16    17    18     1      0.000        5.858       3
   16    17    18    23     1      0.000        0.418       6
   17    18    20    21     1      0.000        0.418       6
   17    18    23    24     1      0.000        0.418       6
   22    23    24    25     1      0.000        5.858       3
   21    22    23    24     1      0.000        5.858       3
   23    22    21    20     1      0.000        5.858       3
   22    21    20    14     1      0.000        5.858       3
   23    24    26    27     1      0.000        5.858       3
   24    26    27    28     1      0.000        5.858       3
   26    27    28    30     1      0.000        0.418       6
   10    11    12    13     1      0.000        1.255       3
   27    28    30    32     1    180.000       33.472       2
   28    30    32    33     1    180.000        0.418       6
   30    32    33    34     1      0.000        0.418       6
```

```
[ dihedrals ]
;  ai    aj    ak    al funct
    2    15     3     1     2    35.264     334.720
    5     4     8     6     2    35.264     334.720
   14    15    20    11     2    35.264     334.720
   15    16     2    14     2    35.264     334.720
   18    17    23    20     2    35.264     334.720
   20    14    21    18     2    35.264     334.720
   23    24    18    22     2    35.264     334.720
   24    26    23    25     2    35.264     334.720
    9     8     2    10     2    35.264     334.720
   11    10    14    12     2    35.264     334.720
   28    30    27    29     2     0.000     167.360
   33    35    34    32     2     0.000     167.360
   30    32    31    28     2     0.000     167.360
```

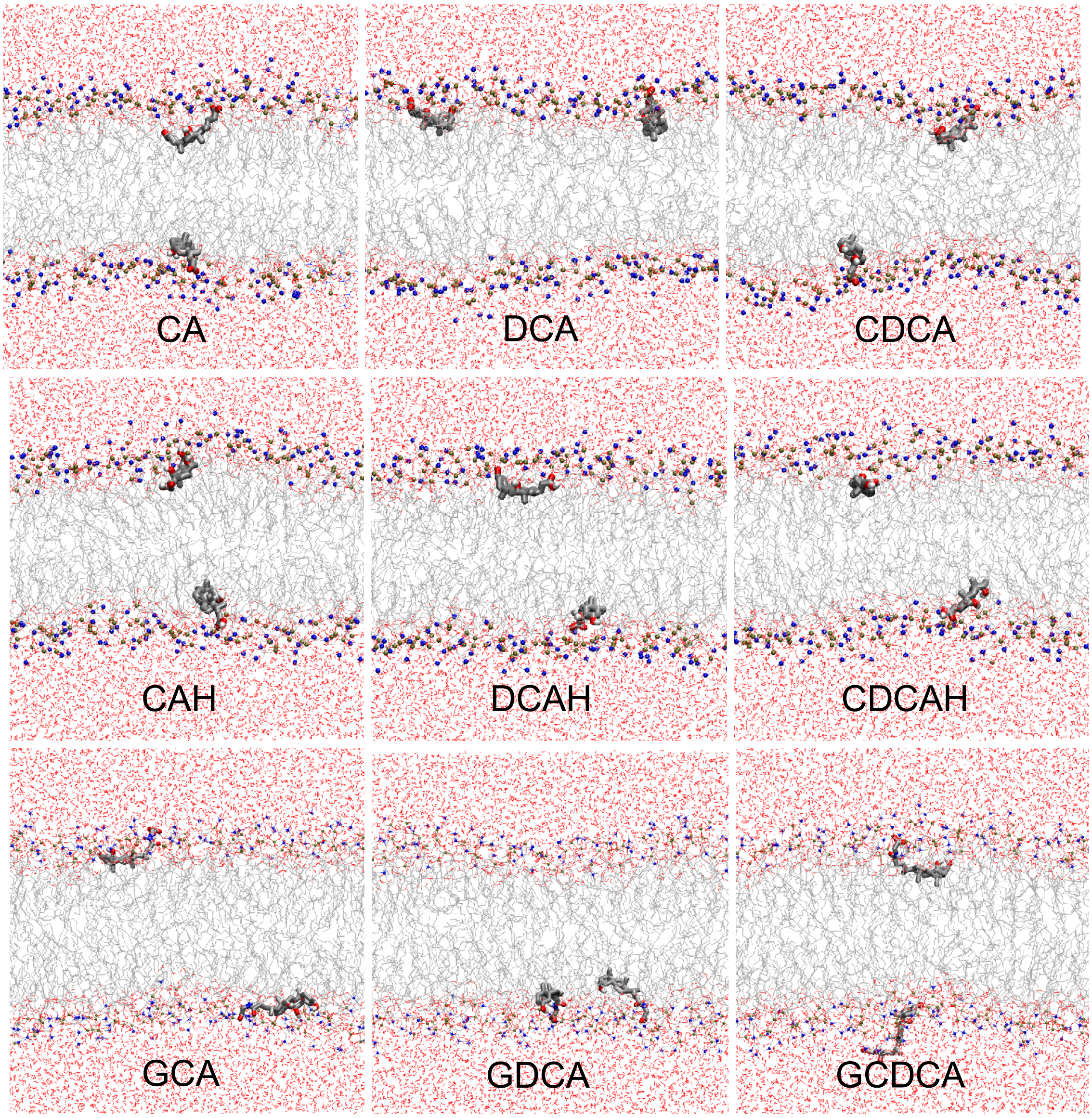

Figure S1 – Final snapshots of the simulations with initial BS location in the water medium. BS molecules are depicted in licorice, while POPC and water molecules are depicted in line style. Atom colors are gray, red, brown, blue and white, for C, O, P, N and H, respectively.

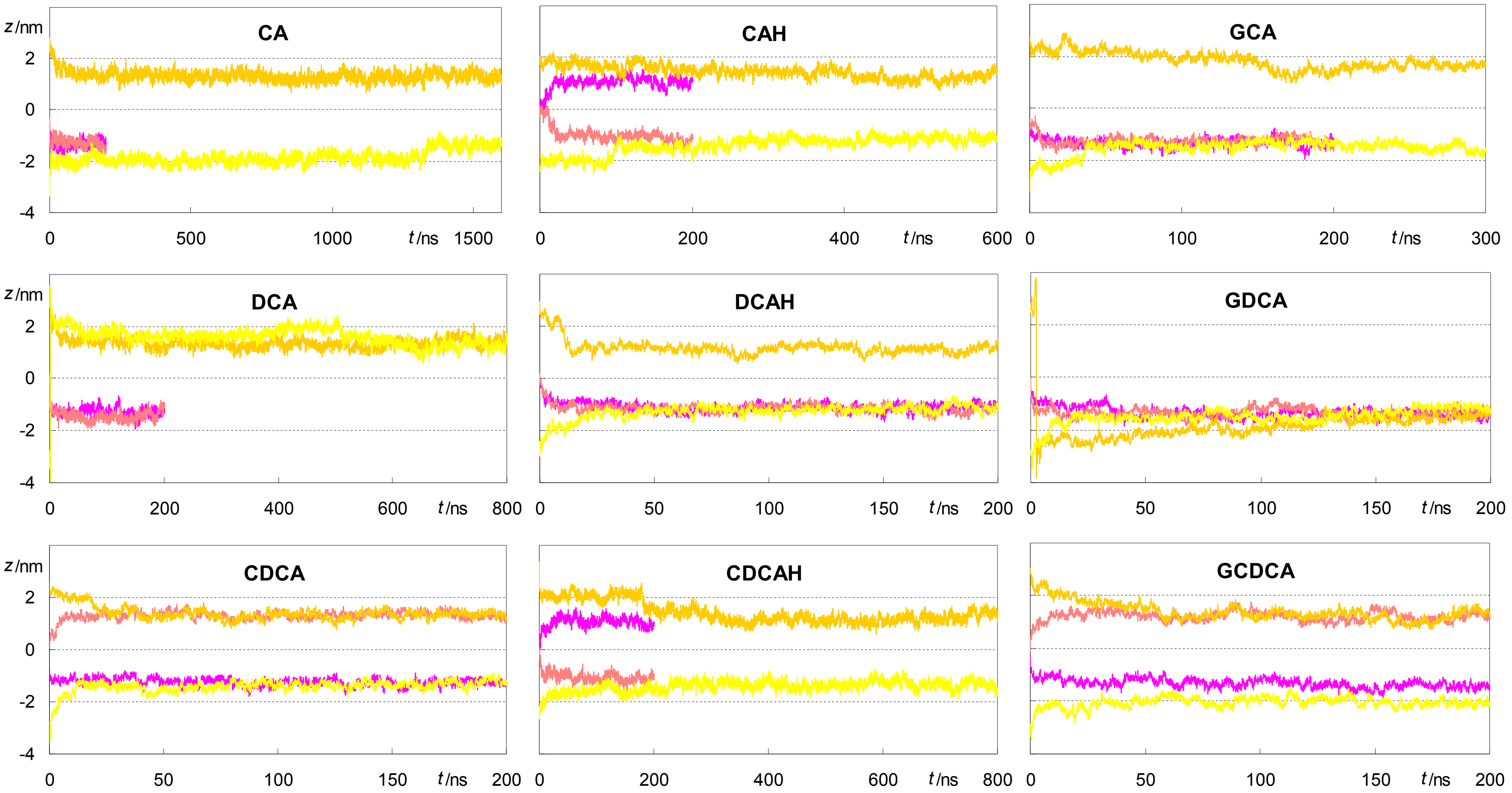

Figure S2 - Time evolution of the BS center of mass *z* coordinate for all simulated molecules across the different systems. Each panel shows the transverse location of the two molecules in the simulation where they were initially placed in the water millieu (gold and yellow), as well as in the one where they were initially placed in the middle of the bilayer (pink and salmon), for a given BS species.

Detailed description of the effect of BS on the POPC headgroup.

Possible perturbation of the headgroup region of the bilayer due to the presence of the BS may be evaluated from the position of the phosphate (P) and choline (N) groups, as well as from the angle between the P-N vector and the bilayer normal (P-N tilt). The results obtained for those parameters are shown in Figures S3 and S4 and Table S1, for all molecules. As shown in Figure S3A, the P-N tilt of the POPC molecules within 0.6 nm from CA is shifted to lower angles (P-N more aligned with the bilayer normal), while that of POPC in close proximity to CAH and GCA is not significantly affected. Changes in the P-N tilt may be originated from alterations in the localization of the P and/or N groups, and those are shown in the middle and bottom panels of Figure S3, respectively. Contrarily to that observed in the P-N tilt, the larger displacement of P and N atoms are observed for the protonated BS (CAH). The choline group from POPC molecules located within 0.6 nm from CAH is at a much deeper position ($\overline{P(z)}$ =1.78 nm) than that of unperturbed POPC bilayers ($\overline{P(z)}$ =1.93 nm). The displacement of the phosphate is in the same direction (1.65 vs 1.83 nm), leading to a smaller change in the resulting P-N tilt. The ionized BS, CA, leads to smaller but opposite displacement of the local P and N groups, thus leading to a large variation in the P-N tilt. Conjugation with glycine tends to attenuate the effects on all parameters.

The results obtained for all parameters for the case of the dihydroxy BS are also shown in Figures S3 and S4. However, even though their effects on POPC headgroup are not identical, they are generally small and no clear patterns can be identified.

For the neutral non-conjugated BS, the plots of Figures S3H and S4H represent the distributions of the phosphate positions in close and intermediate proximity, respectively. CAH leads to the stronger perturbation of the phosphate location, while DCAH and CDCAH lead to similar effects. In this case, a significant effect is observed even at intermediate distances, with all BS leading to a similar perturbation of the POPC bilayer.

The comparison between the effects of all glycine conjugated BS is shown in the right plots of Figures S3 and S4. For those BS, the dihydroxy species lead to more significant effects in the POPC molecules. The choline group is shifted to upper positions in the close proximity of GDCA, while the phosphate is at a lower position near GCDCA. The effect on the phosphate may be due to the formation of a hydrogen bond between

GCDCA amide and the phosphate group (Figure 5). The upward shift of the choline when close to the GDCA may be correlated with the more external location of the carboxylate group for this BS (Figure 3). The origin of this behavior is not clear, but it is tempting to associate it with the presence of the OH group on the same side of the polar tail (DCA) or in the opposite side of the fused rings (CDCA), leading to distinct orientations of the short axis relative to the bilayer normal (Figure 4).

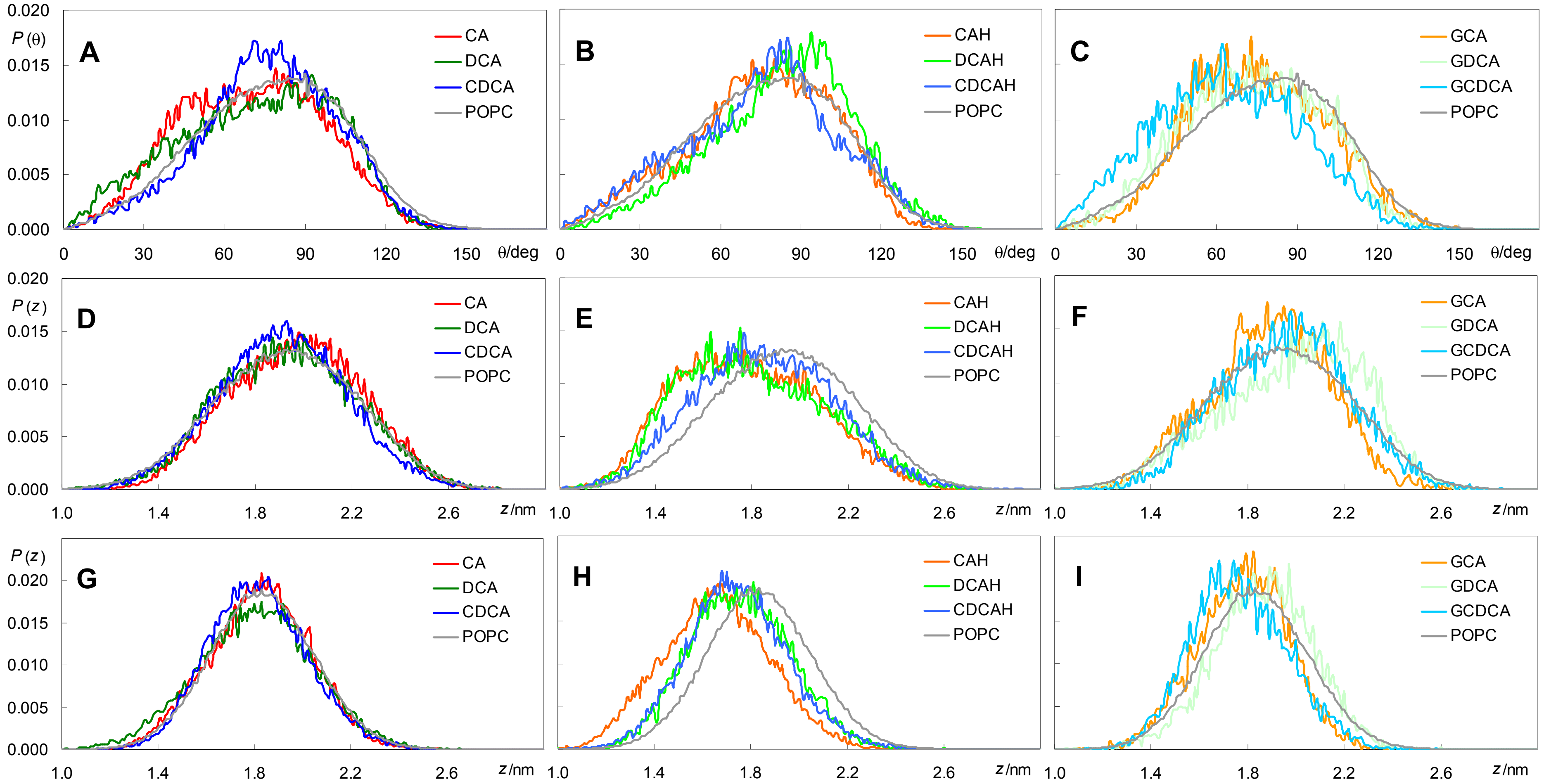

Figure S3 - Distributions of (A-C) POPC P-N tilt relative to the bilayer normal, (D-F) POPC N4 distance relative to the center of the bilayer, and (D-F) POPC P8 distance relative to the center of the bilayer, for lipid molecules at distance $R < 0.6$ nm to the closest BS molecule. The left, middle and right panels concern the effects of unconjugated ionized, unconjugated protonated, and conjugated ionized BS species, respectively. The distributions for pure POPC are also shown in each panel for comparison.

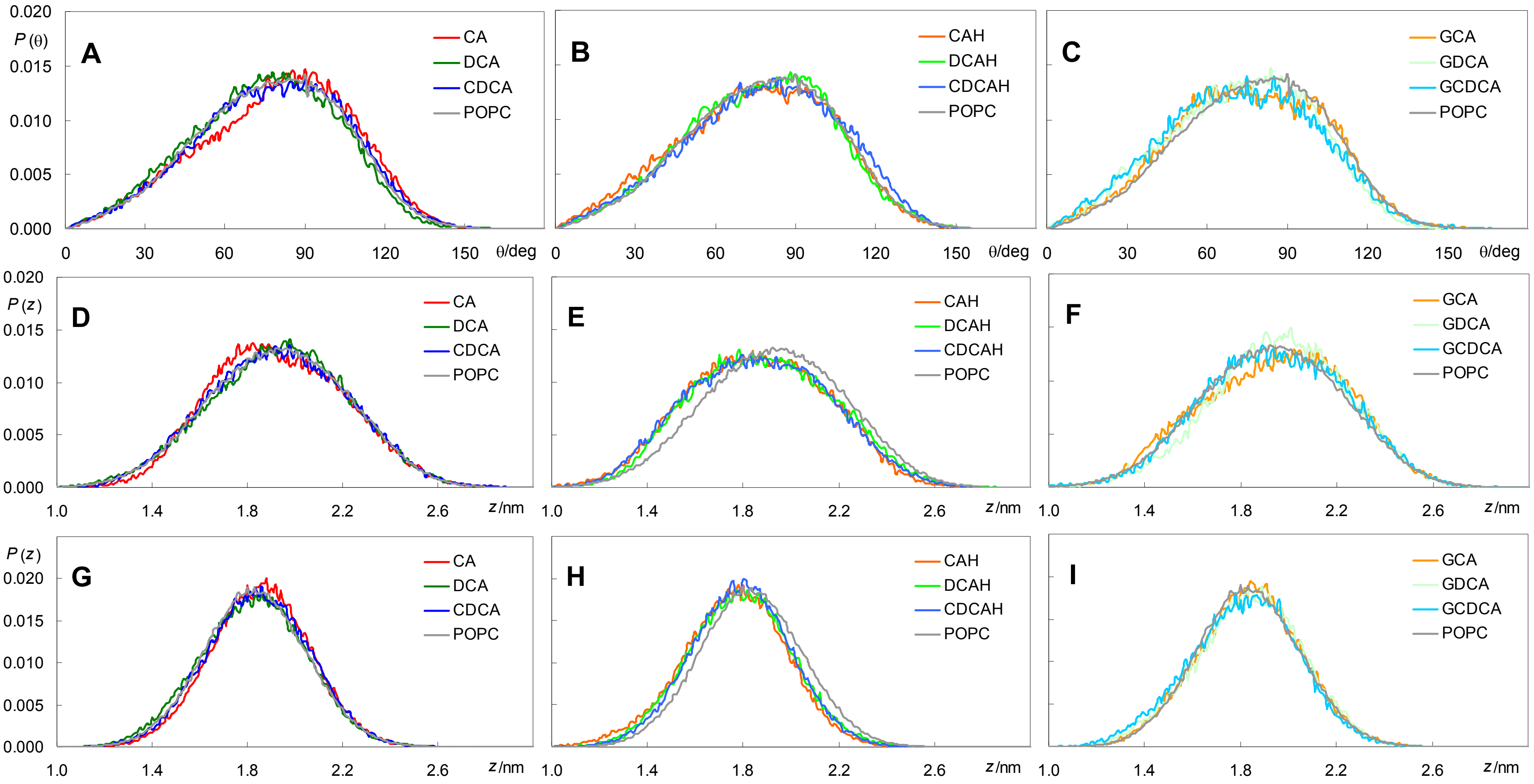

Figure S4 - Distributions of (A-C) POPC P-N tilt relative to the bilayer normal, (D-F) POPC N4 distance relative to the center of the bilayer, and (D-F) POPC P8 distance relative to the center of the bilayer, for lipid molecules at distance 0.6 nm < R < 1.2 nm to the closest BS molecule. The left, middle and right panels concern the effects of unconjugated ionized, unconjugated protonated, and conjugated ionized BS species, respectively. The distributions for pure POPC are also shown in each panel for comparison.

Table S1 – Average tilt angle of the lipid P-N vector, and average position of N and P atoms, for the lipids that are within 0.6 nm from the BS molecules. Values for pure POPC are also shown for comparison.

BS	<Tilt>/deg	<*z*(N)>/nm	<*z*(P)>/nm
CA	70.2	1.97	1.82
DCA	71.9	1.93	1.80
CDCA	76.5	1.91	1.82
CAH	75.0	1.78	1.65
DCAH	82.9	1.80	1.75
CDCAH	74.8	1.85	1.74
GCA	75.4	1.89	1.78
GDCA	73. 4	1.99	1.86
GCDCA	63.9	1.95	1.77
POPC	76.7	1.93	1.83
